# GMPPB‐CDG Results in Lysosomal Dysfunction and Acid Alpha‐Glucosidase Deficiency

**DOI:** 10.1002/jimd.70136

**Published:** 2026-01-19

**Authors:** Carla Damiano, Antonietta Tarallo, Vincenza Gragnaniello, Sandra Strollo, Simona Fecarotta, M. Rosaria Tuzzi, Elena Polishchuk, Sandro Montefusco, Anna Valanzano, Antonia Assunto, Nadia Minopoli, Roberto Della Casa, Roman Polishchuk, Stijn L. M. in 't Groen, Diego Luis Medina, Enrico Bertini, Rosalba Carrozzo, Julia Emmerich, Benedikt Schoser, W. W. M. Pim Pijnappel, Giancarlo Parenti

**Affiliations:** ^1^ Telethon Institute of Genetics and Medicine Pozzuoli Italy; ^2^ Department of Translational Medical Sciences Federico II University Naples Italy; ^3^ European Reference Network for Metabolic Diseases (MetabERN) Naples Italy; ^4^ Department of Pediatrics Erasmus University Medical Center Rotterdam the Netherlands; ^5^ Department of Clinical Genetics Erasmus University Medical Center Rotterdam the Netherlands; ^6^ Center for Lysosomal and Metabolic Diseases, Erasmus University Medical Center Rotterdam the Netherlands; ^7^ Neuromuscular Disorders Research Unit, Bambino Gesù Children's Hospital, IRCCS Rome Italy; ^8^ Unit of Cell Biology and Diagnosis of Mitochondrial Disorders, Laboratory of Medical Genetics, Bambino Gesù Children's Hospital, IRCCS Rome Italy; ^9^ Friedrich‐Baur‐Institut, Neurologische Klinik, Ludwig‐Maximilians‐Universität München Munich Germany; ^10^ Scuola Superiore Meridionale (SSM, School of Advanced Studies) Genomics and Experimental Medicine Program University of Naples, Federico II Naples Italy

**Keywords:** acid alpha‐glucosidase, congenital disorders of glycosylation, GDP‐mannose pyrophosphorylase B deficiency, lysosomal storage diseases, Pompe disease

## Abstract

GDP‐mannose pyrophosphorylase B (GMPPB) deficiency is a congenital disorder of glycosylation due to pathogenic variants of the *GMPPB* gene. GMPPB catalyzes GDP‐mannose synthesis, an early step in multiple glycosylation pathways, including N‐glycosylation, O‐mannosylation, C‐mannosylation, and glycosylphosphatidylinositol‐anchor formation. In fibroblasts (*N* = 3), myoblasts (*N* = 4) and in muscle biopsies (*N* = 4) from a total of 7 GMPPB‐deficient patients we found evidence of glycogen accumulation, both in cytosol and in lysosome‐like vesicles, presence of heterogeneous storage material, and expansion of the lysosomal compartment. Due to the excess of glycogen in cells and tissues, we investigated acid alpha‐glucosidase (GAA) in cultured GMPPB fibroblasts. GAA activity was reduced in GMPPB cells, with an impaired protein maturation and lysosomal localization. Incubation of cells with human recombinant GAA (rhGAA), that is fully glycosylated, showed complete correction of GAA activity, normal processing and lysosomal trafficking, with complete clearance of glycogen storage. These results suggest a secondary impairment of specific lysosomal functions in GMPPB deficiency and add information on the complexity of the pathophysiology of this disorder.

## 1 Introduction

The congenital disorders of glycosylation (CDGs) are a broad family of genetic diseases caused by defects in the synthesis and attachment of glycoprotein and glycolipid glycans [1]. These disorders are due to pathogenic variants in genes encoding enzymes (such as glycosyltransferases and glycosidases) involved in the complex processes of monosaccharide activation, synthesis and remodeling of glycans, sugar nucleotide transporters, or proteins involved in vesicular trafficking, pH homeostasis or Mn^2+^ homeostasis [2]. CDGs are generally systemic diseases and variably affect multiple organs and functions including the central nervous system, muscles, the endocrine system, the eye, the immune system, and coagulation [3].

**FIGURE 1:**
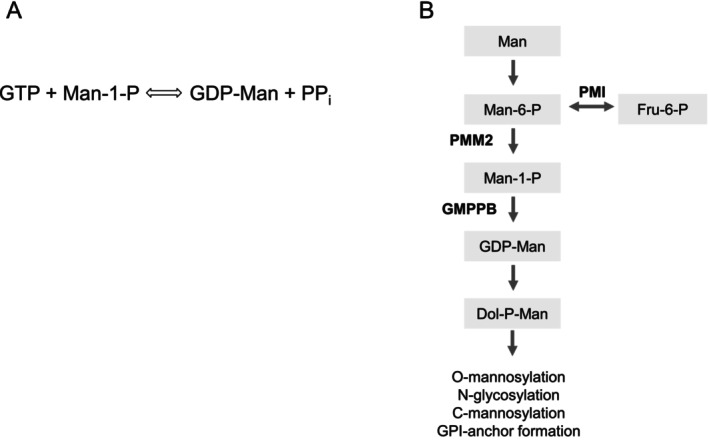
The role of GMPPB in the glycosylation pathway. (A) GMPPB catalyzes GDP‐mannose synthesis. (B) GDP‐mannose acts as a sugar donor and participates in multiple glycosylation pathways, including N‐glycosylation, O‐mannosylation, C‐mannosylation, and glycosylphosphatidylinositol‐anchor formation. The enzymes catalyzing some of the early reactions are indicated in bold. Dol‐P‐Man: dolichol‐phospho‐mannose; Fru‐6‐P: fructose‐6‐phosphate; GDP‐Man: GDP‐mannose; GMPPB: GDP‐mannose pyrophosphorylase B; Man: mannose; Man‐6‐P: mannose‐6‐phosphate; PMI: mannose‐phosphate isomerase; PMM: phospho‐mannomutase 2.

GDP‐mannose pyrophosphorylase B (GMPPB) deficiency, due to pathogenic variants of the *GMPPB* gene [4, 5], is one of these disorders. GMPPB catalyzes the conversion of mannose‐1‐phosphate and guanosine triphosphate into GDP‐mannose and PP_i_ (Figure 1A) in concert with GDP‐mannose pyrophosphorylase A (GMPPA). GDP‐Man is the mannosyl donor for most Man‐containing polymers [6] and participates in multiple glycosylation pathways, including N‐glycosylation, O‐mannosylation, C‐mannosylation, and glycosylphosphatidylinositol (GPI)‐anchor formation [2, 7] (Figure 1B). Thanks to next‐generation sequencing analysis of large cohorts of patients with myopathies, GMPPB deficiency is emerging as a cause of muscle disease that is often overlooked and is probably more prevalent than expected [4, 8, 9].

GMPPB is involved in the glycosylation of alpha‐dystroglycan (ADG) [4]. Thus, GMPPB deficiency is traditionally classified as a dystroglycanopathy, one of several disorders associated with structural abnormalities or defective function of dystroglycan. Dystroglycan is composed of alpha (ADG) and beta (BDG) subunits and is a primary and crucial member of the dystrophin glycoprotein complex (DGC) family in peripheral membranes.

This complex provides a link between proteins located in the extracellular matrix and cytoskeleton proteins, especially in muscles, and has multiple roles in the maintenance of basement membranes, in neuronal migration, and in neuromuscular junction formation. ADG glycosylation is essential for the binding of DGC to extracellular matrix proteins, such as laminin [10]. It has been shown that reduction of ADG glycosylation disrupts these molecular interactions and results in a complex phenotype characterized by muscle damage [11], abnormal neuronal migration, and neurological manifestations [12].

However, as the reaction catalyzed by the GMMPB/A complex is an early and critical step in different types of glycosylation, it is expected that this defect also impacts on glycosylation and processing of other proteins in addition to ADG, and that GMPPB deficiency has effects on multiple cellular pathways and functions. For example, protein glycosylation is critical for some aspects of lysosomal function, such as the correct trafficking of lysosomal enzymes to lysosomes through the mannose‐6‐phosphate receptor pathway [13], and maintenance of lysosomal integrity by mannose‐rich glycosylation of membrane proteins [14]. In this study, we have investigated the effects of GMPPB deficiency on the lysosomal compartment and on one of the lysosomal hydrolases that exerts its function in lysosomes, acid alpha‐glucosidase (GAA), which is involved in glycogen breakdown in these organelles.

## 2 Materials and Methods

### 2.1 Primary Human Cell Cultures

GMPPB and control fibroblasts were available at the Department of Translational Medical Sciences, Federico II University, Naples (GMPPB1, GMPPB2) and at the Bambino Gesu' Hospital, Rome (GMPPB3). Human primary myoblasts and human muscle biopsies (patients 4–7) were provided by the Department of Neurology, Ludwig‐Maximilians‐University, Munich, Germany. The muscle biopsy from patient GMPPB1 was provided by Dr. Chiara Fiorillo (University of Genoa, Italy).

Fibroblasts were grown in Dulbecco's modified Eagle's medium (Invitrogen Corporation, Carlsbad, CA, USA), supplemented with 20% fetal bovine serum (Invitrogen Corporation, Carlsbad, CA, USA), 2 mM L ‐glutamine, 100 U/mL penicillin and 100 μg/mL streptomycin (Invitrogen Corporation, Carlsbad, CA, USA).

Myoblasts were expanded with proliferation medium: High‐Glucose Dulbecco's Modified Eagle's Medium (Invitrogen Corporation, Carlsbad, CA, USA), supplemented with 20% fetal bovine serum (Invitrogen, NY, USA), 10 ng/mL EGF (R&D Systems, Minneapolis, MN, USA), 2 ng/mL β‐FGF (R&D Systems, Minneapolis, MN, USA), 10 μg/mL insulin (Roche Applied Science, Indianapolis, IN, USA) and 1% Penicillin–Streptomycin–Glutamine mix solution (Invitrogen Corporation, Carlsbad, CA, USA). Cells were grown in a 5% CO_2_ humidified atmosphere at 37°C.

Muscle biopsies, fibroblasts, and myoblasts were obtained for diagnostic purposes.

Patients or their legal guardians provided their informed consent to the storage of cells and to their use for research purposes. The experimental procedures involving cells derived from human subjects were conducted in accordance with the principles of the World Medical Association (WMA) Declaration of Helsinki and with the Department of Health and the Human Services Belmont Report.

### 2.2 Electron Microscopy

Muscle biopsies, fibroblasts and myoblasts were fixed in 1% glutaraldehyde in 0.2 M HEPES buffer and post‐fixed in OsO_4_ and uranyl acetate. After dehydration through a graded series of ethanol, the samples were embedded in the Epoxy resin (Epon 812, Sigma–Aldrich) and polymerized at 60°C for 72 h. Thin 60 nm sections were obtained using a Leica EM UC7 microtome. EM images were acquired from thin sections using a FEI Tecnai‐12 electron microscope equipped with a VELETTA CCD digital camera (FEI, Eindhoven, the Netherlands).

### 2.3 Enzyme Activity Assays

Fibroblasts and myoblasts from patients were harvested by trypsinization, resuspended in water, and disrupted by 3 cycles of freezing and thawing. Protein concentration was measured according to the method of Lowry et al. [15]. The enzymatic activity of different lysosomal enzymes was assayed by specific 4‐methylumbelliferyl fluorogenic substrates and stopped by adding 1 mL 0.5 M glycine carbonate buffer, pH 10.7 after specific times of incubation for each reaction.

For GAA activity we used 4‐methylumbelliferyl‐alpha‐D glucopyranoside (Sigma‐Aldrich, St. Louis, MO, USA). Cell homogenates (10 μg proteins) were incubated at 37° for 60 min with 2 mM substrate in 0.2 M acetate buffer pH 4.0 in incubation mixtures of 20 μL.

For β‐galactosidase activity we used 4‐methylumbelliferyl‐β‐D‐galactopyranoside (Sigma‐Aldrich, St. Louis, MO, USA). Cell homogenates (7 μg proteins) were incubated in mixtures of 20 μL with 1 mM substrate dissolved in 0.1 M citrate/0.2 M phosphate buffer, pH 4.0, at 37° for 60 min.

For α‐mannosidase activity we used 4‐methylumbelliferyl α‐D‐mannopyranoside (Sigma‐Aldrich, St. Louis, MO, USA). Cell homogenates (7 μg protein) were incubated at 37° for 30 min with 4 mM substrate dissolved in 0.1 M sodium acetate buffer, pH 4.0, in incubation mixtures of 20 μL.

For β‐glucosidase activity we used 4‐methylumbelliferyl β‐D‐glucopyranoside (Sigma‐Aldrich, St. Louis, MO, USA). Cell homogenates (7 μg protein) were incubated at 37° for 3 h with 5 mM substrate dissolved in 0.1 M sodium acetate buffer, pH 4.2, in incubation mixtures of 20 μL.

After stopping the reactions, the fluorescence was read on a Turner Biosystems fluorometer Modulus 9200 (360 nm excitation, 450 nm emission).

All enzyme assays were performed as technical duplicates for each sample.

### 2.4 Immunofluorescence Analysis

To study the distribution of GAA/LAMP2 human fibroblasts and myoblasts were grown on coverslips, fixed (methanol), permeabilized (phosphate‐buffered saline [PBS]—Triton 0.1%) and blocked (PBS 0.05% saponin, 3% bovine serum albumin) at room temperature for 1 h. Cells were incubated overnight with an anti‐GAA rabbit polyclonal antiserum (PRIMM), and with an anti‐LAMP2 mouse antiserum (Santa Cruz Biotechnology, Dallas, TX, USA). Cells were then incubated with anti‐rabbit IgG and anti‐mouse IgG secondary antibodies (Invitrogen Corporation, Carlsbad, CA, USA) and 40,6‐diami‐dino‐2‐phenylindole (DAPI) (Invitrogen, NY, USA) in blocking solution and finally mounted in Mowiol (Sigma‐Aldrich, St. Louis, MO, USA). Images were taken using a Zeiss LSM700 confocal microscope (Carl Zeiss, Jena, Germany) integrated with the AxioCam MR camera and 63× oil objective. Digital images were captured by using Zeiss AxioVision software and maximum intensity projections were generated using Image J software. At least five images per field were used for quantitative analyses.

### 2.5 Western Blot Analysis

After trypsinization, cells were lysed in water and/or Ripa buffer depending on the specific marker to test (respectively GAA and/or Lamp2). Total protein concentration in cellular extracts was measured by the method of Lowry et al. [15].

Twenty micrograms of proteins from each cell sample were separated by SDS–PAGE and transferred onto nitrocellulose membranes (Amersham, Freiburg, Germany). After blocking with 5% milk, membranes were incubated overnight at 4°C with primary antibodies (anti‐GAA rabbit polyclonal [PRIMM], anti‐Lamp2 mouse [Santa Cruz Biotechnology, Dallas, TX, USA], anti‐Actin mouse [Sigma‐Aldrich, St. Louis, MO, USA], anti‐GAPDH mouse [Invitrogen, NY, USA]). The membranes were washed with Tris buffer saline containing 0.1% Tween‐20 (TBS‐T) and incubated with HRP‐coupled secondary antibodies (Bio‐Rad Laboratories, Hercules, CA, USA) for 1 h at room temperature. Immunoreactive proteins were detected by chemiluminescence (ECL, Amersham, Freiburg, Germany). Images were captured by a CAWOMAT 2000 IR processor or by ChemiDoc XRS+ with Image Lab Software. ImageJ (NIH) or ImageLab software (BioRad Laboratories, Hercules, CA, USA) were used for quantitative analysis. For densitometric quantification, bands were normalized to endogenous reference protein or stain‐free gels.

### 2.6 GMPPB Silencing

For siRNA‐mediated GMPPB silencing, cells were transfected using Lipofectamine LTX and PLUS reagent (Invitrogen, NY, USA) according to the manufacturer's instructions. The siGENOME SMARTpool siRNAs (Dharmacon Thermo Scientific) were transfected to a final concentration of 50 nM and cells were harvested 72 h after transfection.

For studies with the conduritol beta epoxide (CBE) suicide substrate, control fibroblasts were pre‐treated with CBE for 24 h (10 mM). After removing CBE, a sample was harvested for GAA assay, a sample was treated with GMPPB siRNA as indicated above, and a sample was kept untreated for comparison.

### 2.7 Enzyme‐Linked Immune Adsorbent Assays (ELISA)

The levels of GMPPB were measured using a commercial test kit (AMS Biotechnology Ltd., Switzerland, E01M0378) in cell culture supernatants according to the manufacturer's instructions. Briefly, samples and buffer were incubated together with GMPPB‐HRP conjugate in a pre‐coated plate for 1 h. Subsequently, the wells were decanted, washed five times, and incubated with a substrate for 20 min. Finally, a stop solution was added to stop the reaction, and samples were analyzed spectrophotometrically at 450 nm with a SpectraCount apparatus (Packard Bio‐science Company, USA). The GMPPB concentration in each sample was interpolated with a standard curve.

### 2.8 Periodic Acid Schiff Staining

Cells were seeded in chamber slides at a density of 1 × 10^4^/well. After 24 h of culture, cells were fixed with 4% paraformaldehyde for 10 min. Samples were then incubated in 1% periodic acid for 10 min, rinsed in water, and placed in Schiff's reagent for 20 min in accordance with the manufacturer's instructions (Bio‐Optica, Milan, Italy). Then, samples were incubated in fixative solution for 2 min, and finally were incubated in Mayer's Hemalum solution for 3 min and washed under running water for 5 min, allowed to air‐dry. Images were captured by using a Leica DM 5500 microscope (Leica, Wetzlar, Germany). To quantify the number (as fold change with respect to the control cells) of the cells that were PAS‐positive, at least five images were taken of the PAS‐stained slides along with identical fields of view with diastase staining. ImageJ was used to quantify the full area of the cells and the area which was PAS positive for each field.

### 2.9 Glycogen Assay

Cells were assayed for glycogen content using a commercial kit (ab65620, Abcam, Cambridge, UK) according to the manufacturer's instructions. Absorbance was measured at 570 nm using the GloMax Discover System (Promega, Madison, USA).

### 2.10 RNA Isolation and Quantitative Real Time PCR

Total RNA was extracted from cultured fibroblasts and myoblasts using the RNeasy Mini Kit (Qiagen, MD, USA) according to the manufacturer's instructions. cDNA was synthesized from 1 μg of total RNA using the High‐capacity cDNA reverse transcription kit (Thermo Scientific, MA, USA). Quantitative real time PCR (qRT‐PCR) was performed with SYBR Green master mix (Roche, Basel, Switzerland) on a LightCycler 480 System (Roche Diagnostics, Risch‐Rotkreuz, Switzerland). GAA expression was normalized to GAPDH and relative expression was calculated using the 2^−ΔΔCt^ method. Primers sequences were as follows: GAA (Fw: TTCCAACTTCATCAGGGGCT; Rv: GTGCAGGTTGTAGTGTGTGG), GAPDH (Fw: GGGCCAGGTCATCCCTGA; Rv: GCCTGCTTCACCACCTTC).

### 2.11 High‐Content Analysis

Quantitative analysis of the lysosomal area in fibroblasts and myoblasts was performed using a Columbus 2.6.0.127073 apparatus (built at 03:56 on 05/02/19) (PerkinElmer) equipped with Harmony High‐Content Imaging and Analysis Software.

### 2.12 Statistical Analysis

Statistics were performed in GraphPad PRISM software. A two‐tailed, paired and unpaired Student's *t*‐test was performed when comparing the same cell population with two different treatments or cells with different genotypes, respectively. One‐way ANOVA followed by Šídák's post hoc test was used when comparing more than two groups relative to a single factor (treatment). A *p* value of 0.05 or less was considered statistically significant. The results are expressed as the mean ± SD.

## 3 Results

### 3.1 GMPPB Deficiency Is Associated With Pathological Signs of Intracellular Storage

We studied samples of different nature from GMPPB‐deficient patients that had been collected for diagnostic purposes and were already available at our cell or tissue banks, thus avoiding additional invasive procedures, such as skin or muscle biopsies, that might cause unnecessary discomfort to patients.

Specifically, we studied muscle biopsies (*n* = 4) and cultured cell lines (fibroblasts, *n* = 3; myoblast, *n* = 4) from a total of 7 GMPPB patients (Table 1). Samples were derived from patients presenting with variable phenotypes, ranging from severe, early‐onset phenotypes to attenuated, moderate or mild, forms and with different genotypes. All patients had at least one missense mutation of the *GMPPB* gene, none of the patients carrying two null alleles. This finding is consistent with the hypothesis that homozygosity for null variants may be associated with embryonic lethality [16]. Pathogenic variants in other genes involved in myopathies, lysosomal storage diseases and defects of glycogen metabolism were excluded at the time of diagnosis by next‐generation sequencing (NGS) analysis of panels for myopathies and/or lysosomal diseases (see Table S1).

**TABLE 1:** GMPPB patients.

Patient code	Sample(s) studied	Gender	Year of birth	Genotype	Phenotype
GMPPB1	Fibroblasts/muscle biopsy (vastus lateralis)	Male	2007	p.Thr153Ile/p.Gln234*	Severe
GMPPB2	Fibroblasts	Male	2022	p.Thr153lle/p.Arg287Trp	Severe
GMPPB3	Fibroblasts	Female	2002	p.Pro32Leu/p.Arg287Gln	Moderate
GMPPB4	Myoblasts/muscle biopsy (vastus lateralis)	Female	1985	p.Asp27His/p.Gln37*	Moderate
GMPPB5	Myoblasts/muscle biopsy (tibialis anterior)	Male	1977	p.Asp27His/p.Ile2428Thr	Moderate
GMPPB6	Myoblasts/muscle biopsy (vastus lateralis)	Male	1993	p.Asp27His/p.Arg287Trp	Moderate
GMPPB7	Myoblasts (vastus lateralis)	Female	1981	p.Arg287Gln/p.Val357Ile	Mild–Moderate

*Indicates a nonsense mutation leading to a premature stop codon.

**FIGURE 2:**
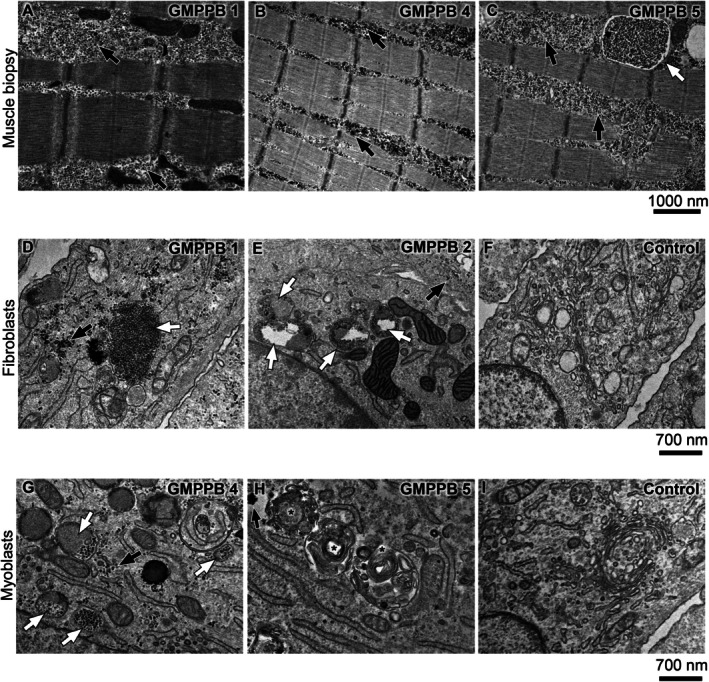
Ultrastructural analysis in GMPPB muscles, cultured fibroblasts and myoblasts. (A–C) Electron microscopy analysis in muscle biopsies show increased amounts of glycogen in the cytosol (black arrows) and in lysosome‐like vesicles (white arrows). We do not show a normal biopsy sample for comparison for ethical reasons, as samples available at our laboratory are only derived from patients with suspected myopathies, thus not to be taken as normal. (D, E) Electron microscopy analysis of GMPPB1 and GMPPB2 fibroblasts, showing glycogen excess in cytosol (black arrows), storage in lysosome‐like structures (white arrows). (F) control fibroblasts. (G, H) Ultrastructural analysis in GMPPB4 and GMPPB5 myoblasts showing glycogen storage in lysosome‐like (white arrows) and multilamellar structures (asterisks). (I) Control myoblasts.

We first performed an electron microscopy (EM) analysis of a quadriceps muscle biopsy, obtained for diagnostic purposes from patient GMPPB1. This patient, described in detail by Fecarotta et al. [17], presented with neurological and cognitive impairment, congenital progressive myopathy, reduced GAA activity in the absence of *GAA* gene pathogenic variants. The most remarkable morphological finding in this sample was the presence of abundant glycogen, that was located both in the cytosol (Figure 2A, black arrows) and in lysosome‐like structures (white arrows).

To confirm the association of such abnormalities with GMPPB deficiency, we extended our studies to muscle biopsies obtained, also for diagnostic purposes, from three additional patients (GMPPB4, GMPPB5, and GMPPB6) and already available at the sample repositories of one of our centers. Similar abnormalities were also found in these samples (Figure 2B,C).

An excess of glycogen was also found in cultured fibroblasts from patients GMMPB1 and GMPPB2 (Figure 2D,E), compared with control fibroblasts (Figure 2F), where it appeared to be localized both in cytoplasm (black arrows) and in lysosome‐like structures (white arrows). Cultured myoblasts from GMPPB patients (Figure 2G,H) also showed similar abnormalities, compared with control myoblasts (Figure 2I), although to a variable extent and in variable association. Multilamellar bodies (asterisks) were also detected both in fibroblasts, and in myoblasts (Figure 2H).

**FIGURE 3:**
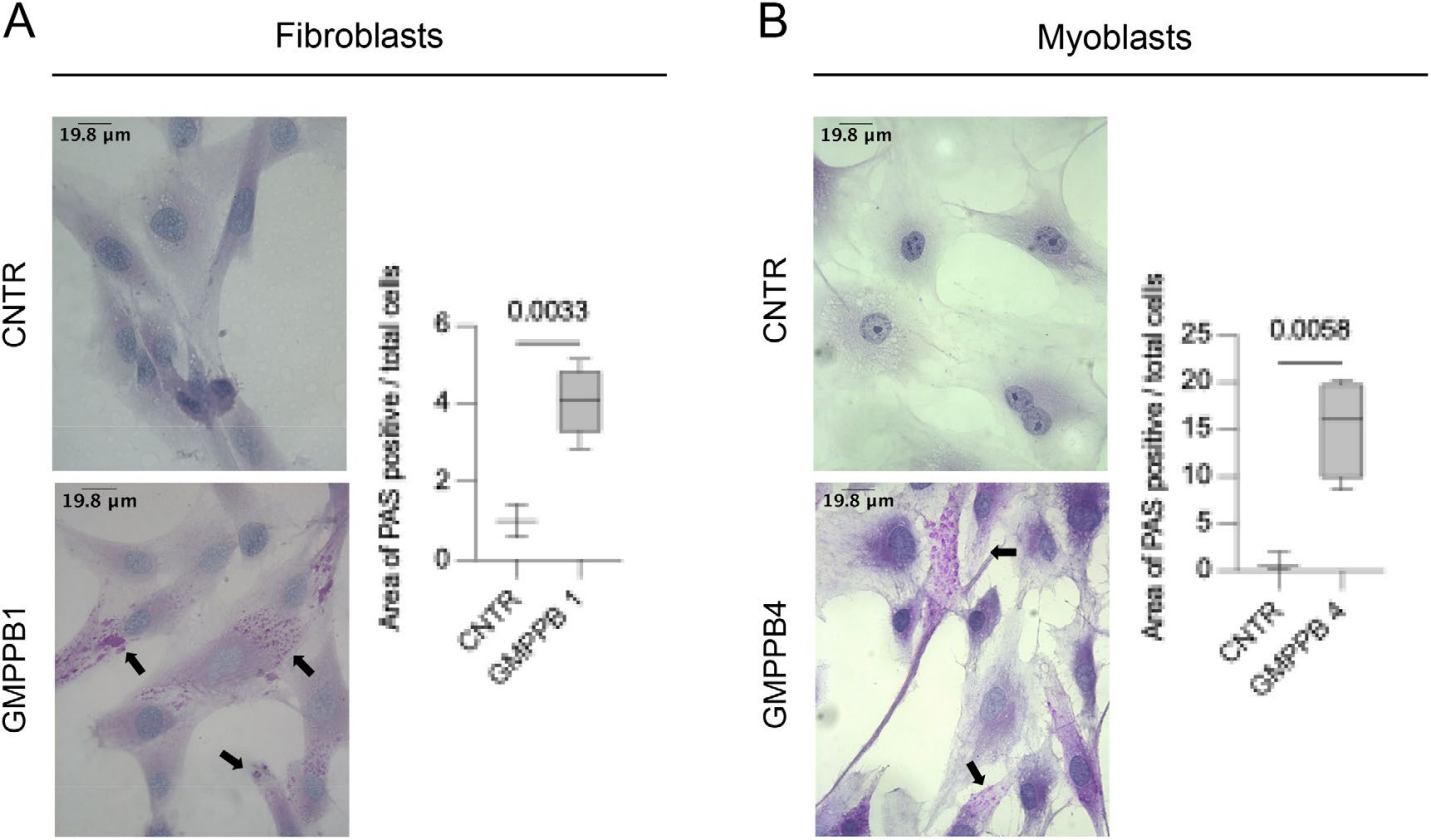
PAS staining in GMPPB fibroblasts and myoblasts.: A periodic acid Schiff (PAS) staining in fibroblasts from patient GMPPB1 (A) and in GMPPB4 myoblasts (B) shows evidence of glycogen excess and PAS‐positive spots (black arrows), with a punctate pattern suggesting lysosomal storage. The analysis of the area occupied by PAS positive structures shows significant increase in both mutant cell lines compared to respective controls. Statistical analysis was performed using a Student's *t*‐test.

To further confirm the presence of increased amounts of glycogen in GMPPB cells, we performed a periodic acid Schiff (PAS) staining. To this purpose, we used fibroblasts from patient GMPPB1 (Figure 3A) and in myoblasts from GMPPB4 (Figure 3B), the ones showing most prominent glycogen accumulation on EM. PAS staining showed increased PAS positivity in both cell lines, mostly with a punctate pattern suggesting lysosomal storage. The analysis of the area occupied by PAS positive structures was significantly increased in both cell lines. Furthermore, a glycogen assay, performed using a commercial kit, showed increased amounts of glycogen in GMPPB1 cultured fibroblast, compared to controls (Figure S1).

**FIGURE 4:**
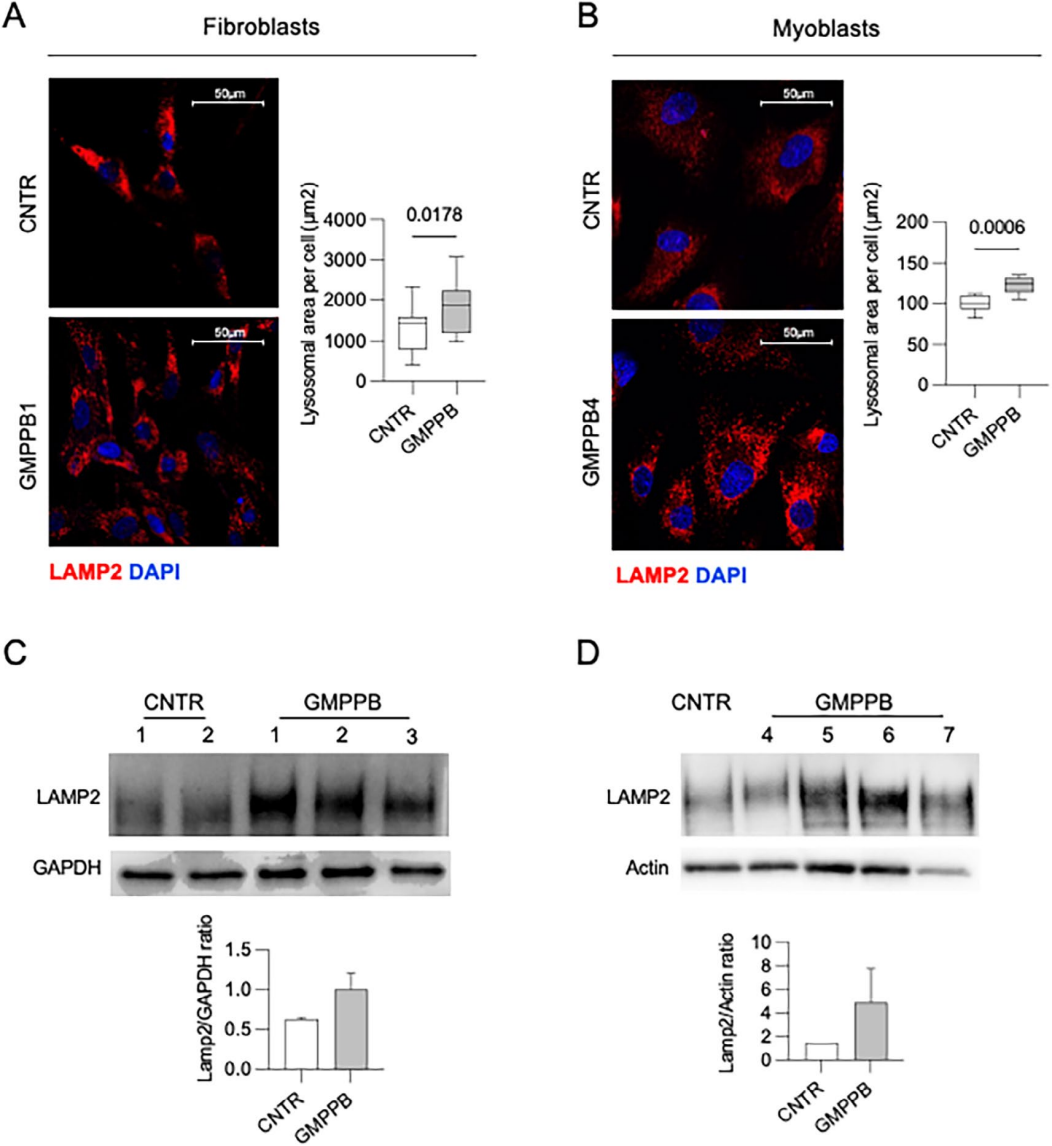
GMPPB cells show lysosomal enlargement and increased amounts of LAMP2. Immunofluorescence of LAMP2 in GMPPB1 fibroblasts (A) and in GMPPB4 myoblasts (B). The LAMP2 signal appears increased in GMPPB cells, as compared to the respective control cells. Confocal 63× images; brightness +45%; contrast +15%. Scale bar 50 μm. A quantitative analysis of the area occupied by lysosomes by high content imaging analysis showed in both cell lines a significant increase in GMPPB cells. A Student's t‐test was applied for statistically significant comparison; *p* values are indicated. Western blot and quantitative analysis of LAMP2 in fibroblasts (C) and myoblasts (D), showing increased amounts of LAMP2 in both cell lines.

Overall, the morphological analysis of multiple samples from GMPPB patients provided evidence of glycogen accumulation, that was peculiarly evident in lysosome‐like structures, and in some samples was associated with multilamellar bodies. The combination of these findings may suggest an involvement of the lysosomal compartment. To further investigate this aspect, we analyzed the amounts and distribution of the lysosomal associated membrane protein 2 (LAMP2), a marker of the late endosomal/lysosomal compartment, in GMPPB1 cultured fibroblasts (Figure 4A) and in GMPP4 myoblasts (Figure 4B).

We observed an increase of the area occupied by the LAMP2 signal, suggesting an expansion of the lysosomal compartment (Figure 4A,B) both in fibroblasts and myoblasts. A quantitative analysis of the area occupied by lysosomes in these two cell lines by using a high‐content imaging platform confirmed the presence of a statistically significant enlargement of the lysosomal compartment. A western blot analysis showed increased amounts of LAMP2 in both cell lines (Figure 4C,D).

### 3.2 GMPPB Cells Show Reduced Acid α‐Glucosidase

In principle, signs of glycogen storage and expansion of the lysosomal compartment may be due to dysfunction of proteins involved in the modulation of the autophagosomal‐lysosomal pathway, to secondary deficiency of specific lysosomal enzymes activities, or both. It is intriguing that lysosomal glycogen storage and impaired autophagy are typical hallmarks of an inborn lysosomal storage disorder, Pompe disease, due to acid α‐glucosidase (GAA) deficiency. Pompe disease, like GMPPB, is characterized by a severe and progressive myopathy that in the early‐onset severe clinical forms is also associated with hypertrophic cardiomyopathy [18]. Therefore, we measured GAA activity in GMPPB1, GMPPB2 and GMPPB3 fibroblasts, and in GMPPB4, GMPPB5, GMPPB6, GMPPB7 myoblasts using a standard artificial fluorogenic substrate.

**FIGURE 5:**
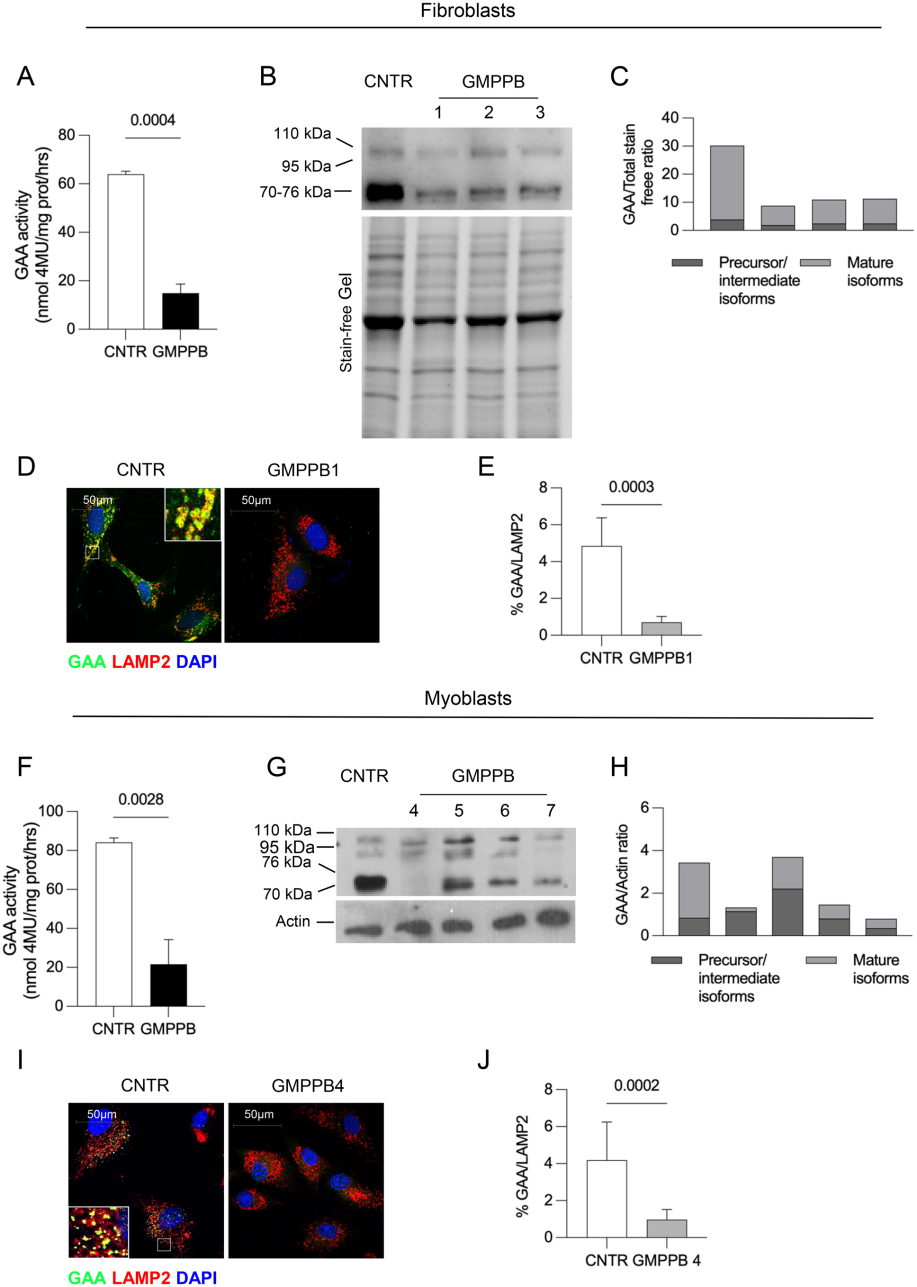
GAA activity, processing and subcellular localization in GMPPB fibroblasts and myoblasts. (A) Mean GAA activity in GMPPB fibroblast cell lines (*N* = 3). GAA activity is significantly reduced compared to control cells. (B) Western blot analysis showing impaired processing of the enzyme in fibroblasts compared to control cells. (C) Quantitative analysis of the GAA isoforms, showing reduced amounts of the enzyme and reduced mature forms. Band intensities are normalized to stain‐free lanes. (D) Immunofluorescence analysis of GAA and LAMP2 co‐localization in GMPPB1 and in control fibroblasts. The inset shows the normal co‐localization of GAA signal with LAMP2 that is not detectable in GMPPB fibroblasts. Confocal 63× images; Scale bar 50 μm. (E) Quantitative analysis of GAA‐LAMP2 colocalization. (F) Mean GAA activity in GMPPB myoblasts (*N* = 4). GAA activity is significantly reduced compared to control cells. (G) Western blot analysis showing reduced amounts of the enzyme in myoblasts compared to control cells. (H) Quantitative analysis of the GAA isoforms, showing reduced amounts of the enzyme and variably reduced mature forms. Band intensities are normalized to Actin. (I) Immunofluorescence analysis of GAA and LAMP2 co‐localization in GMPPB4 and in control myoblasts. The inset shows the normal co‐localization of GAA signal with LAMP2 that is not detectable in GMPPB myoblasts. Confocal 63× images; Scale bar 50 μm. (J) Quantitative analysis of GAA‐LAMP2 colocalization. Statistical analyses were calculated using two‐tailed Student's *t* test.

In these cells GAA activity showed a statistically significant decrease in GMPPB‐deficient cells (Figure 5A, fibroblasts; Figure 5F, myoblasts; Table S2) compared to control cells. Quantitative real‐time PCR analysis showed a trend toward modest and statistically nonsignificant decrease of GAA mRNA transcript in GMPPB fibroblasts and myoblasts (Figure S3). GAA activity and GAA isoforms in the medium of cultured GMPPB fibroblasts (Figure S2), and GAA activity in patient 1 serum were not increased, thus excluding GAA mistrafficking to the secretory pathway and leakage into the extracellular fluid. Other lysosomal enzyme activities (β‐galactosidase; α‐mannosidase; β‐glucosidase) did not show significant changes (Table S2).

To investigate on the reduction of GAA activity in GMPPB cells we analyzed the processing and lysosomal delivery of this enzyme. GAA is synthesized in the endoplasmic reticulum and traffics through the Golgi apparatus, where the 110 kDa precursor enzyme is glycosylated and processed into a 95 kDa intermediate polypeptide and into the 76 and 70 kDa mature lysosomal isoforms [19]. We studied the enzyme maturation by western blot analysis. While in control cells the 76 and 70 kDa mature forms were predominant, in GMPPB cells these polypeptides were relatively reduced compared to precursors (Figure 5B for fibroblasts; 5G for myoblasts), with an altered mature forms/precursors ratio (Figure 5C,H, respectively).

We then studied GAA intracellular localization by immunofluorescence analysis in GMPPB1 fibroblasts and GMPPB4 myoblasts. In these cells GAA signal was hardly detectable compared to the respective control cells, and its co‐localization with LAMP2 signal was significantly reduced (Figure 5D,E for fibroblasts; Figure 5I,J for myoblasts; see also Figure S4).

To further confirm the association between GMPPB deficiency and GAA activity reduction, we silenced *GMPPB* in control fibroblasts. Cells were incubated with a SMARTpool siRNAs and harvested 72 h after transfection. Immune‐reactive GMPPB protein levels and GAA activity were assessed to evaluate the effect of silencing.

**FIGURE 6:**
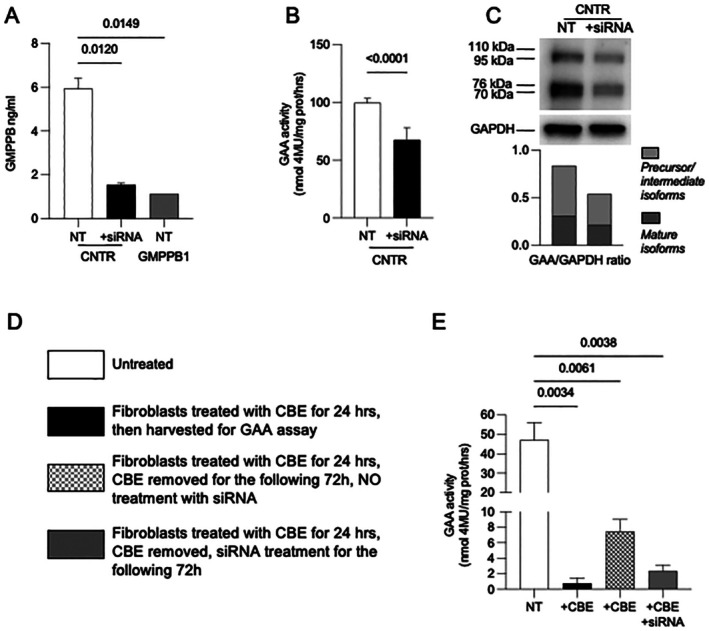
Effect of GMMPB silencing on GAA activity. (A) GMPPB levels in control fibroblasts treated with siRNA silencing are reduced (26% of untreated cells) (black bars) and comparable to those observed in GMPPB1 fibroblasts (gray bars). Statistical assessment performed using one‐way ANOVA, followed by Šídák's multiple comparisons test. (B) GAA activity was decreased silenced cells (black bars), compared to untreated cells (open bars). (C) Western blot analysis of untreated and GMPPB RNA‐silenced control fibroblasts. (D) Experimental design for CBE treatment and GMPPB RNA silencing. (E) Pre‐treatment with CBE followed by GMPPB siRNA treatment results in further reduction of GAA activity (gray bars), compared to cells left untreated after CBE removal (gray and white pattern). Error bars denote mean ± SD. A one‐way ANOVA *t*‐test was applied.

In siRNA‐treated cells, GMPPB levels were substantially reduced (26% of untreated cells) and comparable to those observed in GMPPB1 fibroblasts (Figure 6A). GAA activity in silenced cells was also significantly reduced (Figure 6B), and GAA western blot analysis showed a relative reduction of mature polypeptides (Figure 6C). The presence of some residual GAA activity is not surprising. Because of the relatively long half‐life (days) of the wild‐type enzyme [20], a substantial amount of GAA (synthesized before GMPPB silencing) remains unaffected in a short‐term (72 h) experiment and may in part conceal the effect of *GMPPB* silencing on GAA activity.

To make the effect of GMPPB silencing on GAA activity clearer and more evident, we pre‐treated control fibroblasts with the suicide substrate conduritol beta epoxide (CBE) for 24 h, following the procedures shown in Figure 6D. With this pre‐treatment we could abolish all GAA activity synthesized before GMPPB silencing. In these cells we only detected the activity of GAA synthesized during the 72‐h incubation with GMPPB.

After CBE treatment, part of the cells was harvested, and GAA activity was assayed. In this sample GAA activity was completely abolished (Figure 6E, black bar). Other fibroblasts, after removing CBE, were either left untreated for 72 h (to allow synthesis of new GAA) (Figure 6E, gray and white pattern) or treated for the same time with GMPPB siRNA (Figure 6E, gray). In the cells that had not been exposed to GMPPB siRNA GAA activity was detectable and reflective of the newly synthesized enzyme, while in cells pre‐incubated with CBE and subsequently exposed to GMPPB siRNA, GAA activity was highly decreased.

### 3.3 Recombinant Acid α‐Glucosidase Is Normally Trafficked to Lysosomes and Corrects GAA Activity in GPPB Fibroblasts

Taken together, the reduced maturation and colocalization of the enzyme with LAMP2 suggest an impaired trafficking of GAA to the lysosomal compartment in GMPPB deficiency. To further address this point, we treated GMPPB1 fibroblasts and GMPPB4 myoblasts with recombinant human GAA (rhGAA, alglucosidase alpha) and studied how these cells process this enzyme preparation. rhGAA is used for the enzyme replacement therapy of Pompe disease patients [21, 22] and is manufactured and administered intravenously to patients as a 110 kDa precursor that is fully glycosylated and ready for uptake by the mannose‐6‐phosphate receptor at the plasma membrane of cells. Once internalized by cells, the enzyme enters the endocytic pathway and during its route to lysosomes is processed into the mature and active isoforms.

**FIGURE 7:**
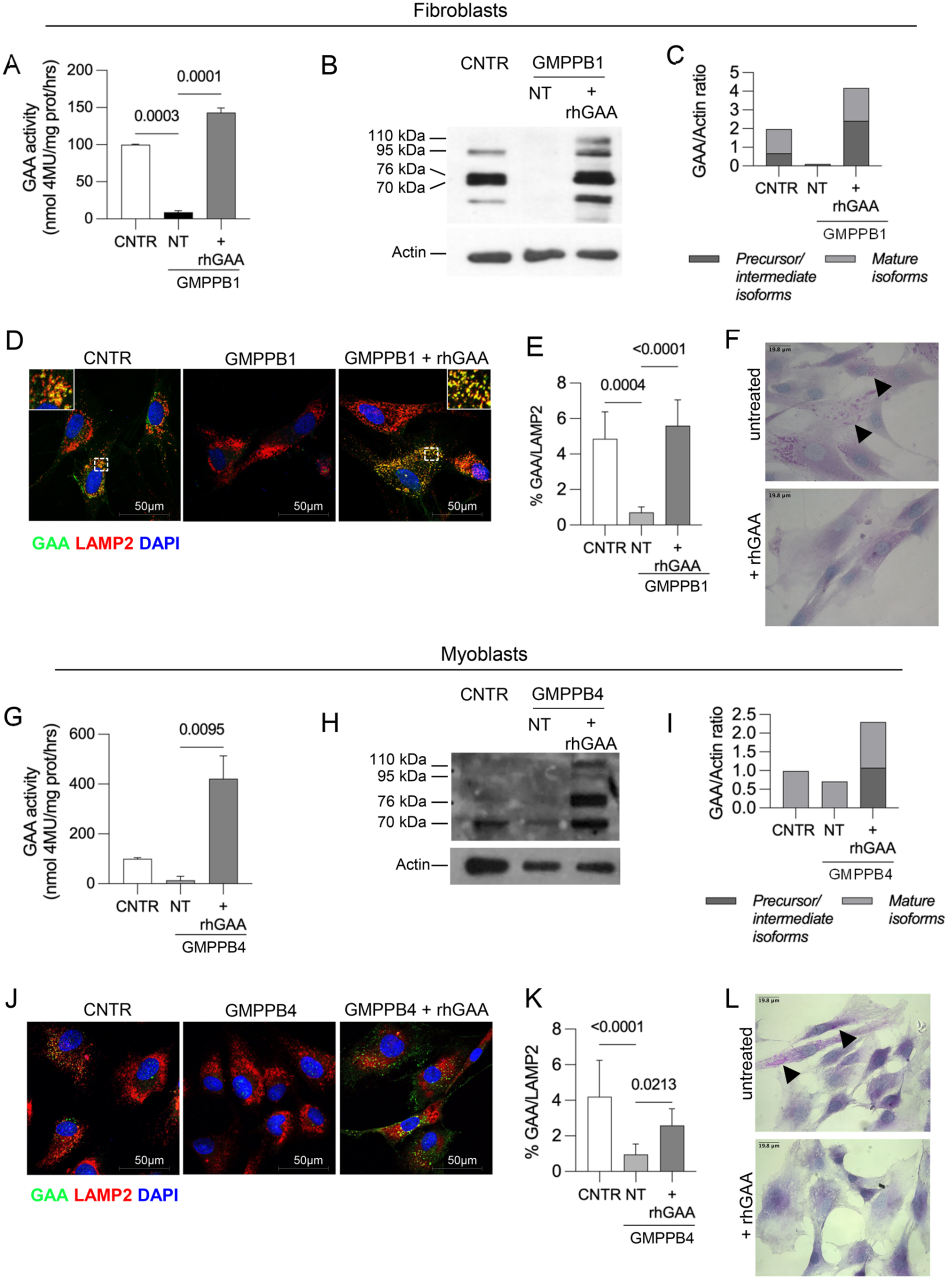
Recombinant acid α‐glucosidase is normally trafficked to lysosomes. (A) Relative GAA activity in GMPPB fibroblasts showing normalization of GAA activity in GMPPB cells treated with rhGAA. GAA activity in control fibroblasts is taken as 100%. (B) Western blot analysis of GAA, showing a normal maturation of the recombinant enzyme in GMPPB1 fibroblasts. (C) Quantitative analysis of the GAA isoforms in the western blot. (D) Immunofluorescence analysis of GAA‐LAMP2 colocalization in untreated and rhGAA‐treated GMPPB fibroblasts. Inset shows the co‐localization of GAA and LAMP2. (E) Quantitative analysis of GAA‐LAMP2 co‐localization from the analysis of five 63× microscopic fields. Scale bar 50 μm. (F) PAS staining of GMPPB1 fibroblasts (top untreated; bottom rhGAA‐treated). (G) Relative GAA activity in GMPPB4 myoblasts showing normalization of GAA activity in cells treated with rhGAA. GAA activity in controls is taken as 100%. (H) Western blot analysis of GAA, showing a normal maturation of the recombinant enzyme in GMPPB4 myoblasts. (I) Quantitative analysis of the GAA isoforms in the western blot. (J) Immunofluorescence analysis of GAA‐LAMP2 colocalization in untreated and rhGAA‐treated myoblasts. Inset shows the co‐localization of GAA and LAMP2. (K) Quantitative analysis of GAA‐LAMP2 co‐localization from the analysis of five 63× microscopic fields. Scale bar 50 μm. (L) PAS staining of GMPPB4 myoblasts (top untreated; bottom rhGAA‐treated). rhGAA treatment results in clearance of PAS‐positive puncta. Magnification, 63×. All *p* values are determined by one‐way ANOVA with Šídák's multiple comparisons.

Both cell lines were incubated for 24 h with 50 μg/mL rhGAA according to published procedures [23]. After treatment, normal GAA enzyme activity was restored (Figure 7A, fibroblasts; Figure 7G, myoblasts) and the pattern of rhGAA processing in fibroblasts was completely normalized by western blot analysis (Figure 7B,C fibroblasts; Figure 7H,I myoblasts).

The colocalization of the enzyme with LAMP2 was comparable to that observed in control untreated cells (Figure 7D,E fibroblasts; Figure 7J,K myoblasts, Figure S5) and significantly improved when compared to GMPPB untreated cells. These data indicate that a normally glycosylated enzyme is able to mature and reach the lysosomal compartment, apparently excluding an effect of GMPPB deficiency on vesicle trafficking.

We also showed that rhGAA treatment of GMPPB cells was able to clear completely the accumulation of PAS‐positive puncta in GMPPB1 fibroblasts (Figure 7F) and in GMPPB4 myoblasts (Figure 7L), indicating that the enzyme reaches the lysosomal compartment, where its action is required, and is active.

## 4 Discussion

This study was prompted by the detection in tissue and cell samples from GMPPB‐deficient patients of peculiar pathological changes that were reminiscent of intracellular storage. Specifically, we found an excess of storage material in muscle preparations and in cultured cells, and signs of expansion of the autophagy‐lysosomal pathway, possibly suggesting a link between this disorder and lysosomal function. These abnormalities were found in samples of different nature from a total of seven patients, which for a rare disorder like GMPPB deficiency represent an adequate cohort. Differences in the expression of the abnormalities in these samples may be explained by considering the genetic and clinical heterogeneity of the patients studied.

Occasional findings of pathological storage in other CDGs have been reported in the literature. Eyskens et al. [24] analyzed postmortem a 5‐month‐old patient with a CDG and found evidence of lysosomal storage by light and electron microscopy, mostly localized in the anterior horn neurons of the spinal cord, with abundant storage material and membranous cytoplasmic bodies. Autopsy records of eight patients with CDG showed myelin‐like fibrillary lysosomal storage in Schwann cells, endo‐ and epineural fibroblasts and in hepatocytes [25]. However, these studies did not provide evidence of impairment of specific lysosomal functions or hypotheses on the mechanisms underlying these peculiar findings in CDGs.

The finding of storage and lysosomal dysfunction may not be totally unexpected in a disorder like GMPPB deficiency. The dysfunction of GMPPB, that catalyzes an early and critical step required for multiple types of glycosylation reactions, is expected to result into aberrant glycosylation of a broad range of proteins (enzymatic, structural, regulatory, etc.) involved in multiple cellular functions, lysosomal function.

Signs of storage and lysosomal dysfunction may also be due to altered glycosylation of specific lysosomal enzymes. In this respect, the excess of structures resembling glycogen on ultrastructural analysis (both in the cytosol and in lysosome‐like vesicles) and the evidence of PAS‐positive material by light microscopy were evocative of a dysfunction of GAA, an enzyme that is involved in the lysosomal breakdown of glycogen. GAA is a glycoprotein with seven N‐glycosylation sites [19] that is synthesized as a 110 kDa precursor and undergoes proteolytic events in the late endosomal/lysosomal compartment [20] and oligosaccharide remodeling with the addition of mannose‐6‐phosphate residues in the Golgi apparatus. Mannose‐6‐phosphate residues are essential for enzyme trafficking and lysosomal delivery and for its processing into the active, mature 76–70 kDa isoforms.

In our study a correlation between GMPPB deficiency and GAA dysfunction was supported by a partial deficiency of this lysosomal activity in GMPPB fibroblasts and myoblasts, by the reduced amounts of GAA polypeptides with defective maturation and lysosomal targeting of the enzyme, by the effect of *GMPPB* gene silencing in control fibroblasts. A possible role of aberrant glycosylation as a determinant of secondary deficiency of GAA was further supported by the experiments with rhGAA which is normally glycosylated and equipped with mannose‐6‐phosphate residues. The data showing full correction of GAA activity, normal maturation by western blot analysis, greater colocalization with LAMP2 and clearance of glycogen storage suggest that GAA deficiency in GMPPB‐deficient cells is due to intrinsic properties of the enzyme, such as glycosylation, rather than a generalized defect of vesicle trafficking in cells.

Although there is an occasional report [26] of reduced activity of multiple lysosomal enzymes in leukocytes from patients affected by PMM2‐CDG, we did not find significant changes in other lysosomal enzyme activities in GMPPB cells. Lysosomal enzymes are variably glycosylated, and it is reasonable to think that they can be variably affected by GMPPB deficiency.

Some pathological clinical similarities between GMPPB and Pompe disease, such as the progressive and debilitating myopathy, are intriguing. It may be speculated that, in addition to the impaired ADG function, secondary GAA deficiency also takes part in the pathophysiology of the myopathy observed in patients with GMPPB deficiency.

CDGs and lysosomal diseases are currently classified as clearly distinct groups of disorders. However, our study suggests that the boundaries between these two classes of inborn metabolic defects may be less defined than traditionally assumed, at least from a biological point of view. Both groups of disorders are multisystemic and involve multiple tissues, organs, and systems, leading to some degree of phenotypic overlap that may be in part explained by the impairment of lysosomal functions.

Protein glycosylation is critical for many cellular pathways and functions. Specifically, GMPPB activity is an early step in multiple types of glycosylation, and it may be expected that a complete deficiency of this activity has devastating effects on cellular functions.

Future research may be aimed at defining the contribution of different cellular pathways to CDGs phenotype. For example, it would be interesting to look at the connections between glycosylation and gluconeogenesis. These processes rely on common metabolic intermediates like glucose‐6‐phosphate and mannose‐6‐phosphate and on common pathways, for example, the hexosamine biosynthesis. There is evidence in the literature that glycogen accumulation may restrict intermediates required for protein glycosylation [27].

It would also be important to characterize in detail the function of the autophagic‐lysosomal pathways in GMPPB‐CDG and in other CDGs. Previous reports suggest secondary dysfunction of this pathways in other disorders of this group, for example in PMM2‐CDG [28] suggesting that involvement of the autophagic‐lysosomal pathway may represent a common mechanism in CDGs.

## Author Contributions

C.D., A.T., and V.G. designed research studies, conducted experiments, analyzed data, and contributed to writing the manuscript, giving equal contribution to all these activities. S.F. participated in the design of the study and in the interpretation of data. M.R.T., S.S., N.M., A.A., A.V. participated in in vitro experiments and cell cultures. E.P., R.P. performed electron microscopy studies. S.M., D.L.M. performed all computerized quantifications and interpreted high‐content analysis data. R.D.C., S.F., E.B., R.C. followed patients and provided clinical information required for interpretation of results. J.E., B.S. cultured and provided cell lines and participated in the interpretation of data. W.W.M.P.P., S.L.M.i.t.G. participated in in vitro experiments, analyzed data, and contributed to the writing of the manuscript. G.P. designed the study, supervised, interpreted the data, and wrote the manuscript. All authors reviewed the manuscript and discussed the work.

## Funding

The authors have nothing to report.

## Ethics Statement

The authors have nothing to report.

## Consent

Informed consent was obtained for diagnostic use and research. Procedures complied with the Declaration of Helsinki.

## Conflicts of Interest

The authors declare no conflicts of interest.

## Supporting information


**Figure S1:** Glycogen assay.
**Figure S2:** GAA activity and western blot analysis in GMPPB1 fibroblast medium.
**Figure S3:** GAA mRNA expression in GMPPB fibroblasts and myoblast.
**Figure S4:** Split‐channel images of GAA and LAMP2 in GMPPB‐deficient cells (related to Figure 5).
**Figure S5:** Split‐channel images of GAA and LAMP2 in GMPPB‐deficient cells (related to Figure 7).

**Table S1:** List of genes analyzed in GMPPB‐deficient patients.

**Table S2:** Lysosomal enzyme activities in GMPPB cells.

## Data Availability

The data that support the findings of this study are available from the corresponding author upon reasonable request.
