# Supplementary material for: GMPPB‐CDG Results in Lysosomal Dysfunction and Acid Alpha‐Glucosidase Deficiency
Source: J Inherit Metab Dis. 2026 Jan 19;49(1):e70136. doi: 10.1002/jimd.70136 (PMC12815487; doi:10.1002/jimd.70136)
Supplement: Supplementary file 1 — Figure S1: Glycogen assay. Figure S2: GAA activity and western blot analysis in GMPPB1 fibroblast medium. Figure S3: GAA mRNA expression in GMPPB fibroblasts and myoblast. Figure S4: Split‐channel images of GAA and LAMP2 in GMPPB‐deficient cells (related to Figure 5). Figure S5: Split‐channel images of GAA and LAMP2 in GMPPB‐deficient cells (related to Figure 7). [file JIMD-49-0-s003.pdf]

Figure S1. Glycogen assay

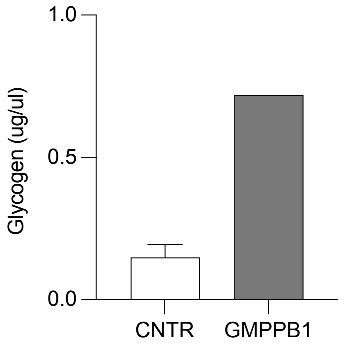

Legend to Figure S1. Glycogen assay measuring glycogen content in control and GMPPB1 using commercial kit, shows increased glycogen levels compared to control fibroblasts.

Figure S2. GAA activity and western blot analysis in GMPPB1 fibroblast medium

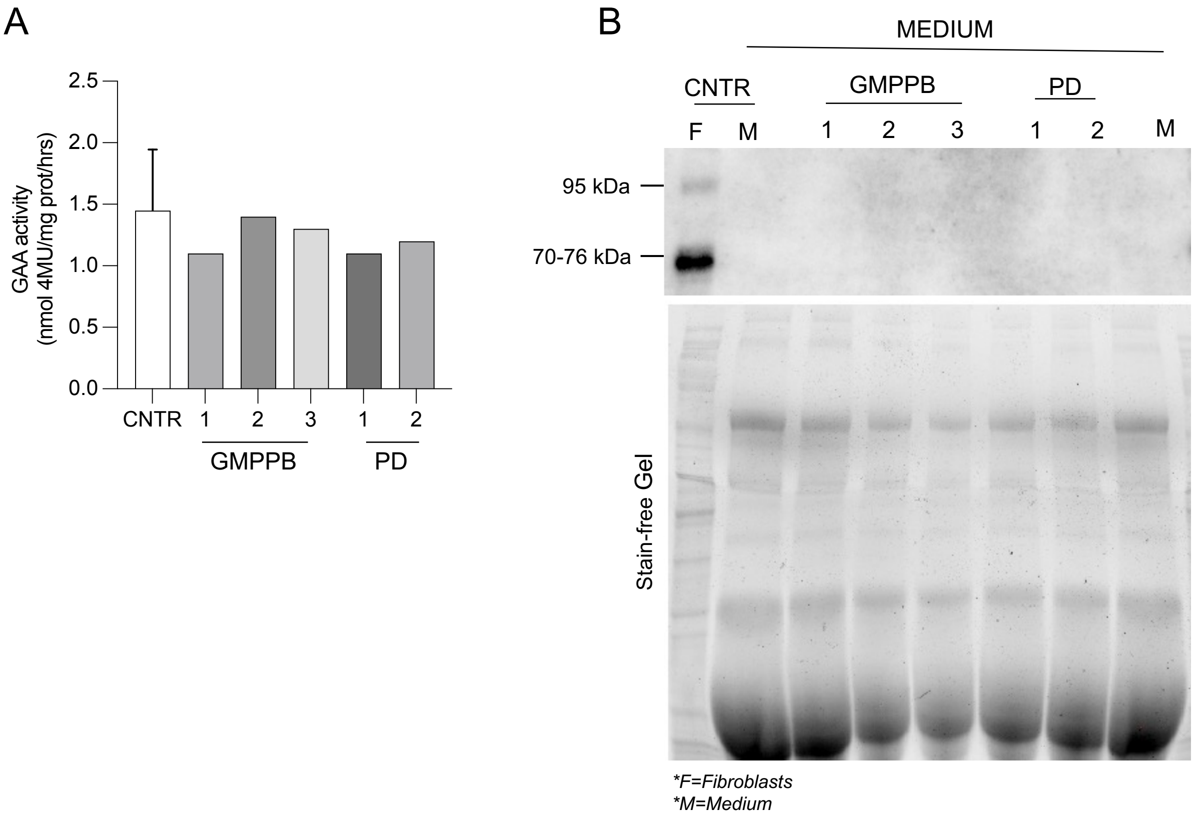

Legend to Figure S2. Assay of GAA activity (A) and Western blot analysis of GAA isoforms in the medium of cultured GMPPB fibroblasts. Both GAA activity and GAA polypeptides are not increased.

Figure S3. GAA mRNA expression in GMPPB fibroblasts and myoblasts

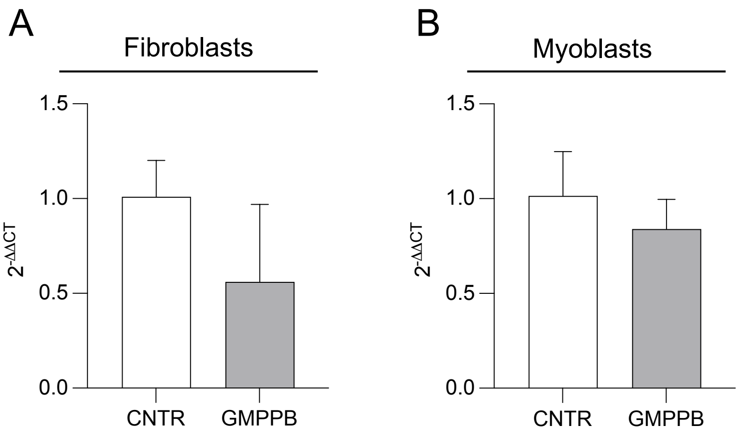

Legend to Figure S3. Quantitative real-time PCR analysis of GAA mRNA levels in GMPPB fibroblasts (A) and myoblasts (B) compared to control cells. GAA expression was normalized to GAPDH and calculated using the $2^{-\Delta\Delta Ct}$ method. Data are expressed relative to controls and shown as mean SD. No statistically significant differences were observed.

Figure S4. Split-channel images of GAA and LAMP2 in GMPPB-deficient cells (related to Figure 5)

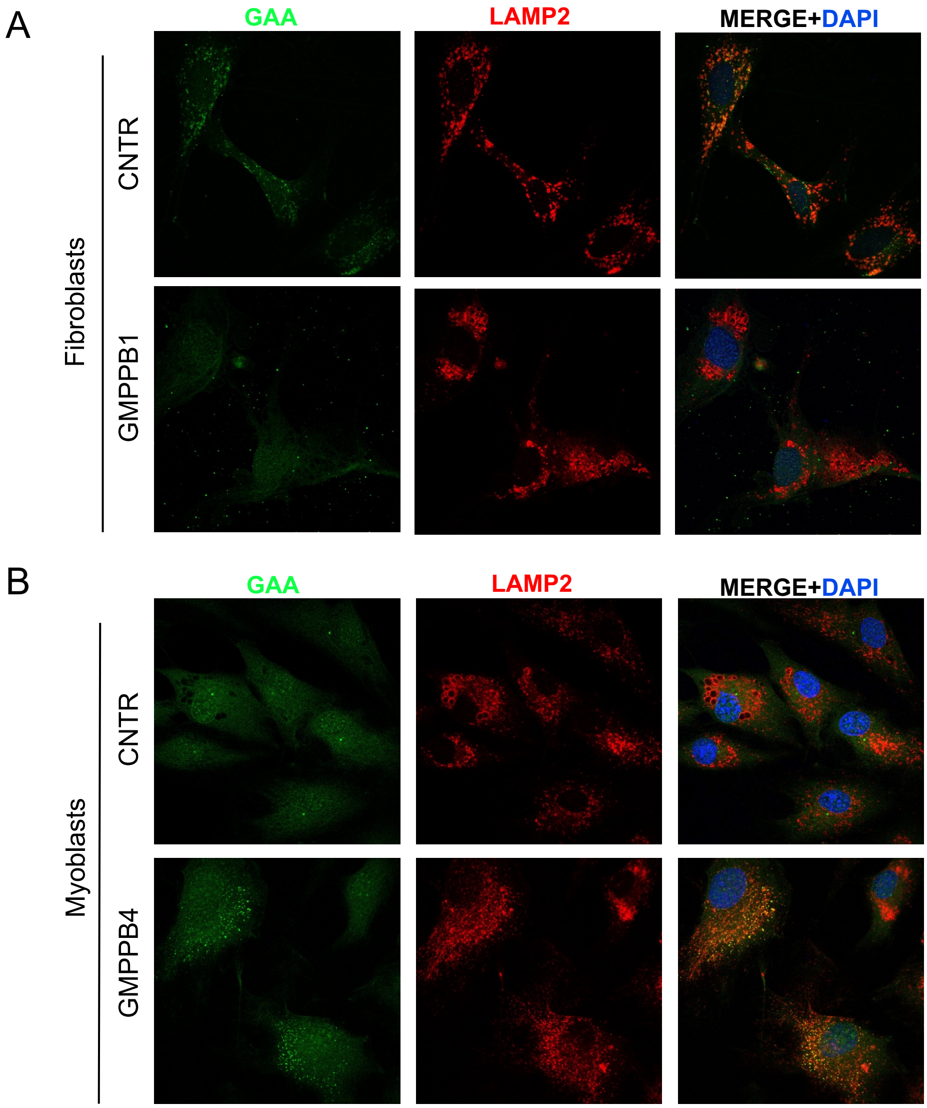

Legend to Figure S4. Split channel immunofluorescence analysis of GAA (green) and LAMP2 (red) in GMPPB cultured fibroblasts (**A**) and myoblasts (**B**). Nuclei are stained with DAPI (blue). Images are related to Figure 5.

Figure S5. Split-channel images of GAA and LAMP2 in GMPPB-deficient cells (related to Figure 7)

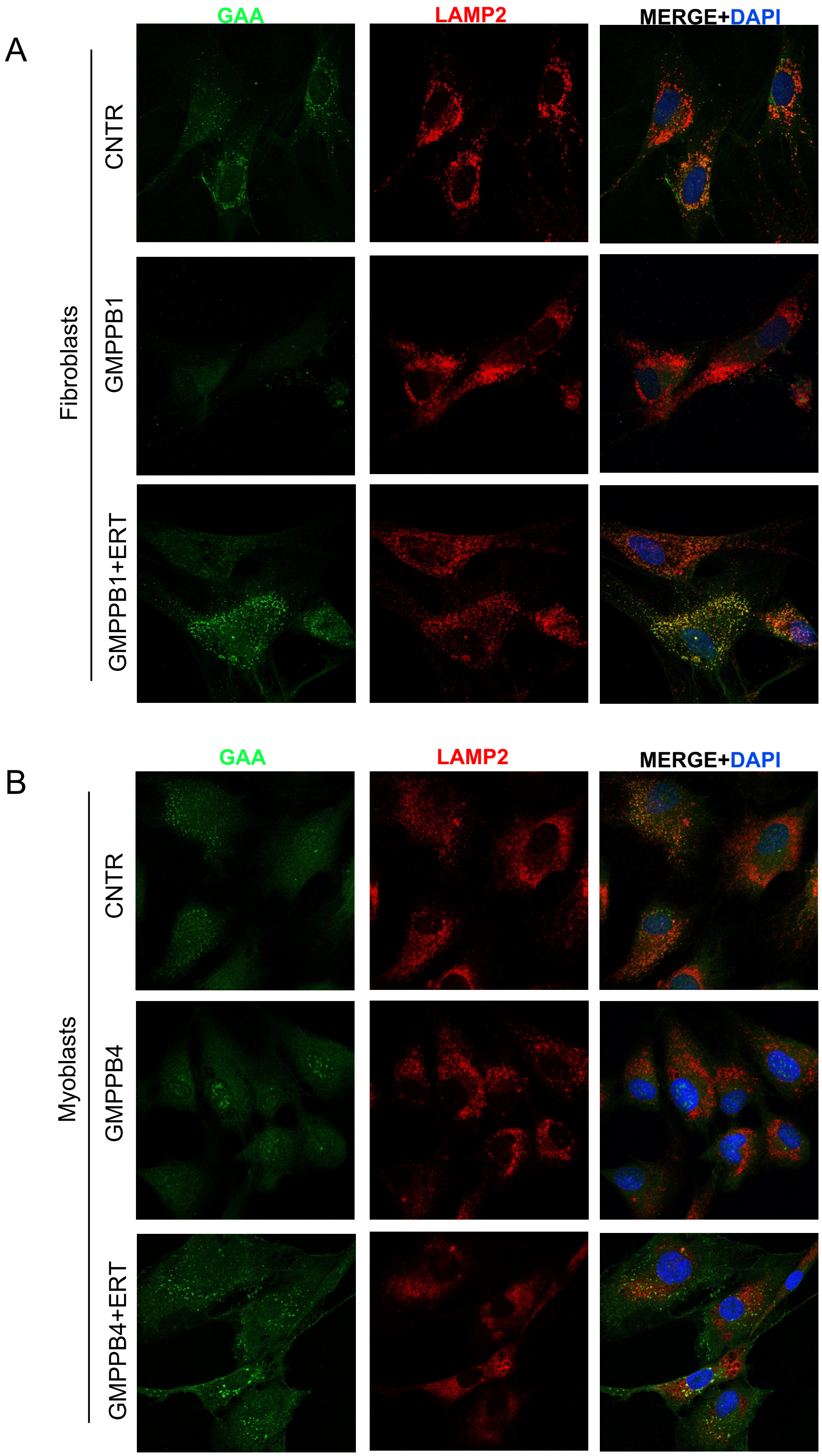

Legend to Figure S5. Split channel immunofluorescence analysis of GAA and LAMP2 in GMPPB cultured fibroblasts (**A**) and myoblasts (**B**). Images are related to Figure 7.
