# Supplementary material for: GMPPB‐CDG Results in Lysosomal Dysfunction and Acid Alpha‐Glucosidase Deficiency
Source: J Inherit Metab Dis. 2026 Jan 19;49(1):e70136. doi: 10.1002/jimd.70136 (PMC12815487; doi:10.1002/jimd.70136)
Supplement: Supplementary file 2 — Table S1: List of genes analyzed in GMPPB‐deficient patients. [file JIMD-49-0-s002.pdf]

Table S1. List of genes analyzed in GMPPB-deficient patients

GMPPB 1

Target ID	Interval	Group(s)				
ACADVL	chr17:7120454-7128426	Group 9				
ACTA1	chr1:229567235-229568872	Group 3				
AGL	chr1:100316588-100387217	Group 9				
ANO5	chr11:22215028-22301321	Group 1	Group 4			
ATP2A1	chr16:28889871-28915775	Group 6				
ATP7A	chrX:77220925-77302077	Group 12				
B3GALNT2	chr1:235613510-235667562	Group 1	Group 2			
BAG3	chr10:121411177-121436804	Group 5				
BIN1	chr2:127806091-127864529	Group 3				
CACNA1S	chr1:201008948-201081477	Group 7	Group 8			
CAPN3	chr15:42646593-42704425	Group 1				
CAV3	chr3:8775552-8787563	Group 1	Group 4	Group 5	Group 6	Group 10
CFL2	chr14:35182060-35183756	Group 3				
CHKB	chr22:51017599-51021220	Group 2				
CNTN1	chr12:41302224-41463847	Group 3				
COL6A1	chr21:47401754-47424662	Group 2				
COL6A2	chr21:47531380-47552476	Group 2				
COL6A3	chr2:238233406-238305470	Group 2				
CPT1B	chr22:51007756-51016354	Group 9				
CPT2	chr1:53662605-53679277	Group 9				
CRYAB	chr11:111779477-111782458	Group 5				
DES	chr2:220283174-220290722	Group 1	Group 5			
DMD	chrX:31132797-33357392	Group 1				
DNAJB6	chr7:157151256-157208802	Group 1				
DNM2	chr19:10824132-10941733	Group 2	Group 3	Group 4		
DOLK	chr9:131707955-131709592	Group 10				
DPM2	chr9:130697990-130700109	Group 2				
DPM3	chr1:155112427-155112816	Group 1				
DYSF	chr2:71681118-71913632	Group 1	Group 4			
EMD	chrX:153607834-153609567	Group 1				
ENO3	chr17:4855114-4860352	Group 9				
FHL1	chrX:135251951-135292194	Group 1	Group 2	Group 5		
FKRP	chr19:47258697-47260205	Group 2				
FKTN	chr9:108337303-108402414	Group 1	Group 2			
FLNC	chr7:128470681-128498587	Group 4	Group 5			
GAA	chr17:78078375-78093140	Group 9				
GBE1	chr3:81539547-81810833	Group 9				
GMPPB	chr3:49759174-49761169	Group 1	Group 2			
GNE	chr9:36216214-36277049	Group 4				
GTDC2	chr3:43121170-43122933	Group 2				
GYG1	chr3:148709417-148744788	Group 9				
GYS1	chr19:49472534-49496379	Group 9				
ISPD	chr7:16131309-16460957	Group 2				
ITGA7	chr12:56078831-56106063	Group 2				
KBTBD13	chr15:65369143-65370540	Group 3				
LAMA2	chr6:129204380-129837502	Group 2				
LAMP2	chrX:119562328-119603034	Group 5				
LARGE	chr22:33562738-34157473	Group 2				
LDB3	chr10:88428438-88492743	Group 4	Group 5	Group 10		
LDHA	chr11:18418084-18428933	Group 9				
LMNA	chr1:156084699-156109640	Group 1	Group 10			
MEGF10	chr5:126666990-126793020	Group 3				
MTM1	chrX:149761066-149840078	Group 3				
MYBPC3	chr11:47353411-47374208	Group 3	Group 10			
MYH2	chr17:10368761-10451247	Group 3				
MYH7	chr14:23882052-23902951	Group 3	Group 4	Group 10		
MYOT	chr5:137206330-137223084	Group 1	Group 4	Group 5		
NEB	chr2:152342263-152589680	Group 3	Group 4			
PDE9A	chr21:44073914-44195413	Group 16				
PFKM	chr12:48499819-48539501	Group 9				
PGAM2	chr7:44102352-44105138	Group 9				
PGK1	chrX:77359827-77381337	Group 9				
PGM1	chr1:64059149-64125356	Group 9				
PHKA1	chrX:71800841-71933738	Group 9				
PLEC1	chr8:144990334-145049547	Group 1	Group 5			
POMGNT1	chr1:46654380-46663503	Group 1	Group 2			
POMT1	chr9:134379595-134398503	Group 1	Group 2			
POMT2	chr14:77743708-77787034	Group 1	Group 2			
PTRF	chr17:40556694-40575125	Group 1				
PYGM	chr11:64514017-64527380	Group 9				
RRM2B	chr8:103220350-103251112	Group 16				
RYR1	chr19:38924459-39078070	Group 3	Group 8			
SEPN1	chr1:26126711-26142219	Group 2	Group 3	Group 5		
SGCA	chr17:48243391-48253222	Group 1				
SGCB	chr4:52890112-52904435	Group 1				
SGCD	chr5:155756576-156186411	Group 1				
SGCG	chr13:23777823-23898690	Group 1				
SLC22A5	chr5:131705654-131729974	Group 9				
SLC25A20	chr3:48895132-48936237	Group 9				
SMCHD1	chr18:2656064-2802560	Group 1				
SUCLA2	chr13:48517495-48575415	Group 16				
SYNE1	chr6:152443560-152949476	Group 1				
SYNE2	chr14:64375856-64692292	Group 1				
TCAP	chr17:37821602-37822372	Group 1	Group 2			
TK2	chr16:66542649-66584196	Group 16				
TMEM43	chr3:14166683-14183305	Group 1				
TNNT1	chr19:55644272-55660584	Group 3				
TNPO3	chr7:128597298-128694834	Group 1				
TPM2	chr9:35682067-35689824	Group 3				
TPM3	chr1:154130104-154164504	Group 3				
TRIM32	chr9:119460011-119461993	Group 1	Group 3			
TTN	chr2:179391728-179682294	Group 1	Group 3	Group 4	Group 5	Group 10
VCP	chr9:35057103-35072360	Group 4				

* Group 1 = Muscular Dystrophies; Group 2 = Congenital Muscular Dystrophies; Group 3 = Congenital Myopathies; Group 4 = Distal Myopathies; Group 5 = Other Myopathies; Group 6 = Myotonic syndromes; Group 7 =Ion Channel Muscle Diseases; Group 8=Malignan hyperthermia; Group 9 = Metabolic Myopathies; Group 10 = Hereditary Cardiomyopathies; Group 12 = Motor Neuron Diseases;

GMPPB2

AAAS	AMACR	ATP5MC3	CAMK4	COA7	DCX	EIF2AK2
AARS1	AMFR	ATP6V0A1	CAMLG	COA8	DDC	EIF2B1
AARS2	AMPD1	ATP6V1A	CAMTA1	COASY	DDHD1	EIF2B2
ABCA1	AMPD2	ATP7A	CAPN1	COG8	DDHD2	EIF2B3
ABCB7	ANG	ATP7B	CAPN3	COL12A1	DECR1	EIF2B4
ABCC9	ANK2	ATP8A2	CASK	COL13A1	DEGS1	EIF2B5
ABCD1	ANKRD1	ATPAF2	CASQ1	COL15A1	DES	EIF2S3
ABHD12	ANKRD2	ATRN	CASQ2	COL25A1	DGAT2	EIF4A2
ABHD16A	ANO10	ATRX	CASR	COL4A1	DGUOK	ELN
ABHD5	ANO3	ATXN1	CAV1	COL4A2	DHCR24	ELOVL1
ACAD9	ANO5	ATXN10	CAV3	COL6A1	DHDDS	ELOVL4
ACADL	ANTXR2	ATXN2	CAVIN1	COL6A2	DHH	ELOVL5
ACADM	ANXA11	ATXN3	CAVIN4	COL6A3	DHTKD1	ELP1
ACADS	AOPEP	ATXN7	CBY1	COLQ	DHX16	EMD
ACADVL	AP1S1	AUH	CC2D2A	COQ2	DHX9	EMILIN1
ACBD5	AP1S2	B3GALNT2	CCDC28B	COQ4	DLAT	ENO3
ACO2	AP4B1	B3GALT6	CCDC78	COQ5	DMD	ENTPD1
ACOX1	AP4E1	B3GNT2	CCDC82	COQ6	DMPK	EPG5
ACTA1	AP4M1	B4GALNT1	CCDC88C	COQ7	DNA2	EPM2A
ACTA2	AP4S1	B4GAT1	CCNF	COQ8A	DNAJA3	ERBB3
ACTB	AP5Z1	BAG3	CCT5	COQ9	DNAJB2	ERBB4
ACTC1	APOA1	BBS1	CD59	COX10	DNAJB4	ERCC4
ACTG2	APTX	BBS10	CDKN1C	COX14	DNAJB6	ERCC5
ACTN2	AR	BBS12	CEP290	COX15	DNAJC12	ERCC6
ACVR1	ARFGEF3	BBS2	CEP41	COX20	DNAJC19	ERCC8
ADAMTS10	ARG1	BBS4	CFL2	COX6A1	DNAJC3	ERGIC1
ADAMTS15	ARHGEF10	BBS5	CHAT	COX6A2	DNAJC5	ERLIN1
ADAR	ARL13B	BBS7	CHCHD10	COX6B1	DNAJC7	ERLIN2
ADCY5	ARL6	BBS9	CHD7	COX8A	DNM2	ETFA
ADCY6	ARL6IP1	BCAP31	CHD8	CP	DNMT1	ETFB
ADGRG1	ARMC9	BCAS3	CHKB	CPLANE1	DNMT3B	ETFDH
ADGRG6	ARPP21	BCKDHB	CHMP1A	CPOX	DOCK3	EWSR1
ADPRS	ARSA	BCS1L	CHMP1B	CPT1C	DOK7	EXOSC3
ADSS1	ARSI	BEAN1	CHMP2B	CPT2	DOLK	EXOSC5
AFG3L2	ARX	BET1	CHMP3	CRAT	DPAGT1	EXOSC8
AGK	ASAH1	BICD2	CHN1	CRLF1	DPM1	EXOSC9
AGL	ASCC1	BIN1	CHP1	CRPPA	DPM2	EYA4
AGPAT2	ASCC3	BIRC2	CHRM3	CRYAB	DPM3	FA2H
AGRN	ASPH	BRAT1	CHRNA1	CSF1R	DRD2	FAH
AGTPBP1	ASXL1	BSCL2	CHRNB1	CSRP3	DRP2	FAM111B
AGXT	ATAD1	BVES	CHRND	CSTB	DSC2	FAM126A
AHCY	ATAD3A	C19orf12	CHRNE	CTBP1	DSG2	FAM20C
AHI1	ATCAY	C1orf194	CHRNG	CTDP1	DSP	FAR1
AHNAK2	ATG5	C1QBP	CHST14	CTNNA3	DST	FARS2
AIFM1	ATG7	C9orf72	CIDEC	CTNS	DSTYK	FASTKD2
AIMP1	ATL1	CA8	CIZ1	CWF19L1	DTNA	FAT2
AIRE	ATL3	CACNA1A	CLCN1	CYC1	DUX4	FBLN5
AKAP9	ATM	CACNA1B	CLCN2	CYLD	DYNC1H1	FBN2
ALDH18A1	ATN1	CACNA1E	CLMP	CYP27A1	DYSF	FBXL4
ALDH3A2	ATP13A2	CACNA1G	CLN3	CYP2U1	EARS2	FBXO28
ALDH5A1	ATP1A1	CACNA1H	CLN5	CYP7B1	EBF3	FBXO38
ALDOA	ATP1A2	CACNA1S	CLN6	DAB1	EBP	FBXO7
ALG13	ATP1A3	CACNA2D2	CLP1	DAG1	ECE1	FDX2
ALG14	ATP2A1	CACNB1	CLPP	DAO	ECEL1	FDXR
ALG2	ATP2B3	CACNB2	CLTCL1	DARS1	ECHS1	FGD1
ALG3	ATP2B4	CACNB4	CNBP	DARS2	EDN3	FGD4
ALK	ATP5F1A	CAD	CNTN1	DCAF17	EDNRB	FGF14
ALPK3	ATP5F1B	CADM3	CNTNAP1	DCAF8	EEF2	FGFR2
ALS2	ATP5F1E	CALR3	COA3	DCTN1	EGR2	FGFR3

GMPPB2

FHL1	GLE1	GSS	KIF1A	MAP3K20	MYH14	NMNAT2
FICD	GLRA1	IBA57	KIF1B	MAPK8IP3	MYH2	NOL3
FIG4	GLRB	IDS	KIF1C	MAPT	MYH3	NOP56
FILIP1	GLRX5	IFIH1	KIF21A	MARS1	MYH6	NOTCH1
FITM2	GLT8D1	IFRD1	KIF26B	MARS2	MYH7	NOTCH2NLC
FKBP10	GM2A	IGFN1	KIF5A	MAT1A	MYH8	NOVA2
FKBP14	GMPPB	IGHMBP2	KIF5C	MATR3	MYL1	NPC1
FKRP	GNAL	ILK	KIF7	MB	MYL2	NPC2
FKTN	GNAO1	IMPDH2	KLC2	MBNL2	MYL3	NPHP1
FLAD1	GNB1	INF2	KLC4	MCM3AP	MYL9	NPPA
FLNA	GNB4	INPP5E	KLHL13	MCOLN1	MYLK	NPTX1
FLNB	GNE	INPP5K	KLHL40	MDM1	MYLK2	NR4A2
FLNC	GOLGA2	INSR	KLHL41	MECR	MYLPF	NRAP
FLVCR1	GOSR2	IQGAP3	KLHL7	MED11	MYMK	NRG1
FLVCR2	GPAA1	IRF2BPL	KLHL9	MED12	MYMX	NRG3
FMR1	GPC3	IRF6	KMT2B	MED25	MYO18B	NRTN
FOLR1	GPD1L	ISCA1	KPNA3	MED27	MYO9A	NT5C2
FOXG1	GPT2	ISCA2	KY	MEGF10	MYOD1	NTRK1
FOXL2	GRID2	ISCU	L1CAM	MET	MYOT	NUBPL
FOXRED1	GRIN1	ISLR2	L2HGDH	MFF	MYOZ1	NUDT2
FRMD5	GRM1	ITGA7	LAMA1	MFN2	MYOZ2	NUP155
FTL	GRN	ITM2B	LAMA2	MGME1	MYPN	NUP54
FUCA1	GSN	ITPR1	LAMA4	MIB1	NADK2	NUP88
FUS	GSX2	ITPR3	LAMA5	MICU1	NAGA	NUS1
FXN	GTPBP2	JAG1	LAMB2	MKKS	NAGLU	OBSCN
FXR1	GYG1	JAG2	LAMP2	MKS1	NALCN	OFD1
GAA	GYS1	JPH1	LARGE1	MLIP	NARS1	OPA1
GAB1	HACD1	JPH2	LARS2	MMACHC	NDRG1	OPA3
GAD1	HACE1	JPH3	LAS1L	MMADHC	NDUFA11	OPHN1
GALC	HADH	JSRP1	LDB3	MME	NDUFAF1	OPTN
GALT	HADHA	JUP	LDHA	MOGS	NDUFAF2	ORAI1
GAMT	HADHB	KARS1	LETM1	MORC2	NDUFAF3	P4HA1
GAN	HARS1	KAT6B	LGALSL	MPDU1	NDUFAF4	PABPN1
GARS1	HARS2	KBTBD13	LGI4	MPV17	NDUFAF5	PALMD
GATAD1	HCN4	KCNA1	LIFR	MPZ	NDUFB11	PANK2
GBA	HEXA	KCNA2	LIG3	MRE11	NDUFB3	PARK7
GBA2	HEXB	KCNA5	LIMS2	MRPL3	NDUFB9	PAX6
GBE1	HIBCH	KCNC3	LITAF	MRPL44	NDUFS1	PAX7
GBF1	HINT1	KCND3	LMNA	MRPS25	NDUFS2	PAXBP1
GCDH	HK1	KCNE1	LMNB1	MSC	NDUFS3	PCCA
GCH1	HMBS	KCNE2	LMNB2	MSTN	NDUFS4	PCCB
GDAP1	HMGCR	KCNE3	LMOD1	MSTO1	NDUFS6	PCDH12
GDAP2	HNRNPA1	KCNH2	LMOD3	MTAP	NDUFV1	PCK2
GDNF	HNRNPA2B1	KCNJ10	LMX1B	MTCL1	NDUFV2	PCYT2
GEMIN5	HNRNPDL	KCNJ11	LONP1	MTFMT	NEB	PDCL3
GFAP	HOXA1	KCNJ18	LPIN1	MTM1	NEFH	PDE10A
GFER	HOXD10	KCNJ2	LRIF1	MTMR14	NEFL	PDE2A
GFPT1	HPCA	KCNJ5	LRP12	MTMR2	NEK1	PDGFB
GGPS1	HPDL	KCNJ6	LRP4	MTO1	NEK9	PDGFRB
GIPC1	HPRT1	KCNK3	LRSAM1	MTPAP	NEMF	PDHA1
GJA1	HRAS	KCNMA1	LYRM7	MTRFR	NEXN	PDHX
GJA5	HS2ST1	KCNN2	LYST	MTTP	NFATC2	PDK3
GJB1	HSPB1	KCNQ1	MACF1	MUSK	NFU1	PDSS1
GJB3	HSPB3	KCNQ2	MADD	MVK	NGF	PDSS2
GJC2	HSPB8	KCNQ3	MAFB	MYBPC1	NGLY1	PDXK
GK	HSPD1	KCTD17	MAG	MYBPC3	NHLRC1	PDYN
GLA	HSPG2	KDM5C	MAGEL2	MYF6	NIPA1	PET100
GLB1	HTRA2	KIAA1109	MAMDC2	MYH1	NKX2-1	PEX1
GLDN	IARS2	KIDINS220	MAN2B1	MYH11	NKX6-2	PEX10

GMPPB2

PEX11B	POMGNT1	RMND1	SEPSECS	SMAD3	TAF1	TNNI3
PEX12	POMGNT2	RNASEH1	SEPTIN9	SMAD4	TAF15	TNNT1
PEX13	POMK	RNASEH2A	SERAC1	SMCHD1	TAFAZZIN	TNNT2
PEX14	POMT1	RNASEH2B	SETX	SMN1	TAMM41	TNNT3
PEX16	POMT2	RNASEH2C	SGCA	SMPD4	TANGO2	TNPO2
PEX19	POPDC3	RNF13	SGCB	SMPX	TARDBP	TNPO3
PEX2	POR	RNF170	SGCD	SMYD1	TBC1D23	TNXB
PEX26	POU4F1	RNF216	SGCE	SNAP25	TBC1D24	TOP3A
PEX3	PPARG	RNF220	SGCG	SNAP29	TBCD	TOR1A
PEX5	PPOX	RNU7-1	SGCZ	SNAPC4	TBCE	TOR1AIP1
PEX6	PPP2R2B	ROBO3	SGO1	SNORD118	TBK1	TPI1
PEX7	PPP3CA	RORA	SGPL1	SNTA1	TBP	TPK1
PFKM	PRDM12	RPGRIP1L	SH3BP4	SNX14	TBX22	TPM1
PFN1	PRDM16	RPH3A	SH3TC2	SOD1	TBX5	TPM2
PGAM2	PRDX3	RRM2B	SHMT2	SORD	TCAP	TPM3
PGAP1	PREPL	RS1	SHQ1	SOX10	TCF21	TPP1
PGK1	PRG4	RTN2	SIGMAR1	SPART	TCTN1	TPR
PGM1	PRICKLE1	RUBCN	SIL1	SPAST	TCTN2	TRAPPC11
PHKA1	PRKAG2	RXYLT1	SKI	SPATA5L1	TCTN3	TRAPPC2L
PHKB	PRKCG	RYR1	SLC12A6	SPEG	TDP1	TRAPPC6B
PHOX2A	PRKN	RYR2	SLC16A1	SPG11	TDP2	TRDMT1
PHYH	PRKRA	RYR3	SLC16A2	SPG21	TECPR2	TRDN
PI4KA	PRNP	SACS	SLC17A5	SPG7	TFAM	TREX1
PIEZO2	PRPH	SALL4	SLC18A2	SPR	TFG	TRIM2
PIGS	PRPS1	SAMD9	SLC18A3	SPTAN1	TGFB1	TRIM32
PIK3R1	PRRT2	SAMD9L	SLC19A3	SPTBN2	TGFB2	TRIM54
PIK3R4	PRUNE1	SAMHD1	SLC1A3	SPTBN4	TGFB3	TRIM63
PIK3R5	PRX	SAR1B	SLC1A4	SPTLC1	TGFBR1	TRIP4
PINK1	PSEN1	SARS1	SLC20A2	SPTLC2	TGFBR2	TRPA1
PIP5K1C	PSEN2	SARS2	SLC22A5	SPTSSA	TGM6	TRPC3
PITRM1	PSMB8	SBDS	SLC25A1	SQSTM1	TH	TRPV1
PKP2	PSMC3	SBF1	SLC25A15	SRD5A3	THAP1	TRPV4
PLA2G6	PTRH2	SBF2	SLC25A20	SRF	THG1L	TSEN2
PLD3	PTS	SCARF2	SLC25A26	SRPK3	TIA1	TSEN34
PLEC	PUM1	SCN10A	SLC25A3	SS18L1	TIMM22	TSEN54
PLEKHG5	PUS1	SCN11A	SLC25A4	SSPN	TIMM50	TSFM
PLIN1	PYGM	SCN1A	SLC25A42	STAC3	TIMM8A	TSPOAP1
PLN	PYROXD1	SCN2A	SLC25A46	STIM1	TINF2	TTBK2
PLOD1	QDPR	SCN3B	SLC29A3	STIM2	TK2	TTC19
PLOD2	RAB3GAP2	SCN4A	SLC2A1	STUB1	TLN1	TTC8
PLP1	RAB7A	SCN4B	SLC30A10	STXBP1	TLN2	TTI1
PMM2	RAD21	SCN5A	SLC30A9	SUCLA2	TMEM106B	TTN
PMP2	RAF1	SCN8A	SLC33A1	SUCLG1	TMEM126B	TTPA
PMP22	RAPSN	SCN9A	SLC35A3	SUFU	TMEM138	TTR
PMPCA	RARS2	SCO1	SLC39A14	SUOX	TMEM151A	TUBA1A
PMPCB	RBCK1	SCO2	SLC44A1	SURF1	TMEM216	TUBA4A
PNKD	RBM10	SCYL1	SLC4A10	SVBP	TMEM231	TUBB2A
PNKP	RBM20	SCYL2	SLC52A1	SVIL	TMEM237	TUBB2B
PNPLA2	RBM7	SDHA	SLC52A2	SYN2	TMEM240	TUBB3
PNPLA6	REEP1	SDHAF1	SLC52A3	SYNE1	TMEM43	TUBB4A
PNPLA8	REEP2	SDHD	SLC5A6	SYNE2	TMEM63C	TWIST1
PNPT1	RELN	SDR39U1	SLC5A7	SYNGAP1	TMEM65	TWIST2
PODXL	RET	SEC31A	SLC6A3	SYNJ1	TMEM67	TWNK
POGLUT1	RETREG1	SECISBP2	SLC6A5	SYNM	TMEM70	TYMP
POLD1	RFC1	SELENOI	SLC6A9	SYNPO2	TMPO	UBA1
POLG	RFXANK	SELENON	SLC9A1	SYT1	TNNC1	UBA5
POLG2	RHOBTB2	SEMA3C	SLC9A3R1	SYT14	TNNC2	UBAP1
POLR3A	RINT1	SEMA3D	SLC9A6	SYT2	TNNI1	UBQLN2
POLR3B	RIPK4	SENP7	SLK	TACO1	TNNI2	UBQLN4

GMPPB2

UBR4
UBTF
UCHL1
UNC13A
UNC45B
UNC50
UNC80
UQCC2
UQCC3
UQCRB
UQCRC2
UQCRQ
USP14
USP8
VAC14
VAMP1
VAMP2
VAPB
VCL
VCP
VIPAS39
VLDLR
VMA21
VPS11
VPS13A
TRPM3
VPS13D
VPS16
VPS33B
VPS37A
VPS41
VPS4A
VRK1
VWA1
VWA3B
WARS1
WARS2
WASHC5
WDPCP
WDR45
WDR45B
WDR48
WDR73
WDR81
WFS1
WNK1
WWOX
XK
XRCC1
YARS1
YARS2
YIF1B
YY1
ZBTB42
ZC4H2
ZFHX2
ZFR
ZFYVE26
ZFYVE27
ZIC3
ZMPSTE24
ZNF423
ZNF526
ZNF592

MYOPATHIEN / MUSKELDYSTROPHIEN

- ☐ 089.12 **Myopathien/Muskeldystrophien – Gesamtpanel** I *244 Gene siehe www.mgz-muenchen.de*
- ☐ 165.10 **Exom / Exom Trio**

Nach Leitsymptomen

- ☐ 975.02 **Leitsymptom Myalgien, Muskelkrämpfe, ggf. moderate CK-Erhöhung** I *ANO5, CAPN3, COL6A1, COL6A2, COL6A3, DMD, FKRP, GAA, GLA, MLIP, PYGM*
- ☐ 974.01 **Leitsymptom CK-Erhöhung, isoliert** I *ANO5, CAPN3, CAV3, CPT2, DAG1, DMD, DYSF, GAA, LAMP2, MLIP, RYR1*
- ☐ 043.01 **Leitsymptom Kontrakturen und/oder Rigid Spine** I *26 Gene siehe www.mgz-muenchen.de*
- ☐ 037.08 **Muskelschwäche, Säuglings- bis Kindesalter, Floppy Infant** I *136 Gene siehe www.mgz-muenchen.de*
- ☐ 045.05 **Muskelschwäche, Erwachsenenalter** I *108 Gene siehe www.mgz-muenchen.de*

Muskeldystrophien

- ☐ **Muskeldystrophie Duchene / Becker (Dystrophinopathie)**
  - ☐ 932.00 Stufe 1: DMD-Dosisanalyse
  - ☐ 020.00 Stufe 2: DMD-Punktmutationsanalyse
- ☐ 247.00 **Muskeldystrophien, Gliedergürteltyp / LGMD (ohne Duchenne/Becker)** I *ANO5, CAPN3, DYSF, FKRP, SGCA, SGCB, SGCD, SGCG*
- ☐ 249.00 **Muskeldystrophien, kongenital, Bethlem/Ullrich (Kollagen-VI-assoziiert)** I *COL6A1, COL6A2, COL6A3*
- ☐ 031.03 **Muskeldystrophien, kongenital, Dystroglykanopathien** I *15 Gene siehe www.mgz-muenchen.de*
- ☐ 251.00 **Muskeldystrophien, kongenital, Gesamtpanel** I *B3GALNT2, B4GAT1, CHKB, COL12A1, COL6A1, COL6A2, COL6A3, CRPPA, DAG1, DNM2, FHL1, FKBP14, FKRP, FKTN, GMPPB, ITGA7, LAMA2, LARGE1, LMNA, PIEZO2, POMGNT1, POMGNT2, POMK, POMT1, POMT2, RXYLT1, SELENON, TCAP, TNXB*
- ☐ 750.00 **Muskeldystrophie, Skapuloperoneale Syndrome** I *CAPN3, CAV3, DES, FKRP, GAA, MYH7, SGCA, TRPV4, VCP*
- ☐ 022.01 **Muskeldystrophie, Typ Emery-Dreifuss** I *EMD, FHL1, LMNA, SYNE2*

Okulopharyngeale Muskeldystrophie

- ☐ 934.00 **Muskeldystrophie, okulopharyngeal** I OPMD (*PABPN1*-Repeat)
- [illegible]

Myopathien

- ☐ 824.00 **Myopathie, axial, adult-onset, dominant** I *CAPN3, FHL1, RYR1*
- ☐ 574.01 **Myopathie, distal / Einschlusskörper-Myopathie** I *DES, GNE, HNRNPA1, LDB3, MYH2, MYH7, MYOT, SQSTM1, VCP*
- ☐ 575.01 **Myopathie, Myofibrilläre** I *BAG3, CRYAB, DES, DNAJB6, FHL1, FLNC, LDB3, MYOT, PYROXD1*
- ☐ 834.00 **Myopathie, Vakuoläre** I *CASQ1, DES, DNAJB6, GAA, GNE, GYG1, LDB3, MATR3, MYH7, TCAP, TIA1, VCP*
- ☐ 827.01 **Myopathie, proximal, mit Myalgien / adult** I *ANO5, CAPN3, CAV3, FKRP, GAA, MLIP, ORAI1, PNPLA2, PYGM, SCN4A, STIM1*
- ☐ 028.01 **Myopathie, kongenital, Zentronukleäre** I *BIN1, DNM2, MTM1, MTMR14, RYR1*
- ☐ 026.04 **Myopathie, kongenital, Zentronukleäre / Core / Nemaline** I *ACTA1, BIN1, CCDC78, CFL2, DNM2, KBTBD13, KLHL40, LMOD3, MEGF10, MTM1, MTMR14, MYH7, MYPN, NEB, RYR1, SELENON, TNNT1, TPM2, TPM3*
- ☐ 000.00 **Myopathie, Viszerale** I *ACTG2*

Mitochondriale Myopathien

- ☐ 973.00 **CPEO (Chronisch Progressive Externe Ophthalmoplegie), nukleäre Gene** I *DGUOK, DNA2, DNM2, MGME1, OPA1, POLG, RNASEH1, RRM2B, SLC25A4, SPG7, TK2, TYMP*
- △ 000.00 **CPEO, sporadisch – mtDNA Deletion/en** [2]
- ☐ 752.01 **Myopathie, mitochondrial, nukleäre Gene** I *CHKB, DGUOK, DNA2, ETFDH, FDX2, FLAD1, HADHA, HADHB, ISCU, MGME1, OPA1, POLG, PUS1, RNASEH1, SLC25A32, SLC25A4, TK2,TMEM126B, TWNK, TYMP*
- ☐ 699.00 **Myopathie, mitochondrial, mtDNA Sequenzanalyse** [2]
- △ 000.00 **Myopathie, mitochondrial, mtDNA – Deletion/en** [2]

[2] *vorzugsweise Muskel-DNA*

Metabolische Myopathien

- ☐ 025.05 **Metabolische Myopathie / Fettsäureoxidationsstörung / Myalgien / Rhabdomyolyse** I *ABHD5, ACADM, ACADS, ACADVL, AGL, AMACR, AMPD1, ANO5, CPT1A, CPT2, DMD, DYSF, ETFA, ETFB, ETFDH, FDX2, FKRP, GAA, HADH, HADHA, HADHB, ISCU, LPIN1, MLIP, OBSCN, PFKM, PGK1, PNPLA2, PYGM, RYR1, SLC22A5, SLC25A20, TANGO2*
- ☐ 978.00 **Morbus Pompe** I *GAA*
- ☐ 966.00 **McArdle Disease** I *PYGM*
