# Supplementary material for: GMPPB‐CDG Results in Lysosomal Dysfunction and Acid Alpha‐Glucosidase Deficiency
Source: J Inherit Metab Dis. 2026 Jan 19;49(1):e70136. doi: 10.1002/jimd.70136 (PMC12815487; doi:10.1002/jimd.70136)
Supplement: Supplementary file 3 — Table S2: Lysosomal enzyme activities in GMPPB cells. [file JIMD-49-0-s001.pdf]

Table S2. Lysosomal enzyme activities in GMPPB cells

	Fibroblasts				Myoblasts				
	GMPPB			*CONT (range)*	*GMPPB*				*CONT*
	1	*2*	*3*		*4*	*5*	*6*	*7*	
**Alpha-glucosidase**	19.21	12.62	12.95	64.4 ± 22.9	6.22	28.84	16.80	34.52	84.15
**Beta-galactosidase**	224.3	216.0	256.3	408.0 ± 117	133.87	208.21	208.91	241.30	301.64
**Alpha-mannosidase**	95.73	117.9	197.1	172.0 ± 62	76.87	91.86	71.30	65.74	46.77
**Beta-glucosidase**	24.3	26.8	13.8	104.8 ± 60,9	12.32	12.98	9.4	23.71	12.32
